# Locus-specific LINE-1 expression in clinical ovarian cancer specimens at the single-cell level

**DOI:** 10.1038/s41598-024-54113-w

**Published:** 2024-02-21

**Authors:** Anna Perkiö, Barun Pradhan, Fatih Genc, Anna Pirttikoski, Sanna Pikkusaari, Erdogan Pekcan Erkan, Matias Marin Falco, Kaisa Huhtinen, Sara Narva, Johanna Hynninen, Liisa Kauppi, Anna Vähärautio

**Affiliations:** 1 Research Program in Systems Oncology, Research Programs Unit, Faculty of Medicine, University of Helsinki, 00290 Helsinki, Finland; 2 Institute of Biomedicine and FICAN West Cancer Centre, University of Turku and Turku University Hospital, 20521 Turku, Finland; 3 Department of Obstetrics and Gynecology, University of Turku and Turku University Hospital, 20521 Turku, Finland; 4 Foundation for the Finnish Cancer Institute (FCI), Helsinki, Finland; 5 Present Address: Sandra and Edward Meyer Cancer Center, Weill Cornell Medicine, New York, NY USA; 6 Present Address: New York Genome Center, New York, NY USA; 7 Present Address: Faculty of Medicine and Health Technology, Tampere University, Tampere, Finland

**Keywords:** Cancer, Cancer genetics, Genetics, Gene expression

## Abstract

Long interspersed nuclear elements (LINE-1s/L1s) are a group of retrotransposons that can copy themselves within a genome. In humans, it is the most successful transposon in nucleotide content. L1 expression is generally mild in normal human tissues, but the activity has been shown to increase significantly in many cancers. Few studies have examined L1 expression at single-cell resolution, thus it is undetermined whether L1 reactivation occurs solely in malignant cells within tumors. One of the cancer types with frequent L1 activity is high-grade serous ovarian carcinoma (HGSOC). Here, we identified locus-specific L1 expression with 3′ single-cell RNA sequencing in pre- and post-chemotherapy HGSOC sample pairs from 11 patients, and in fallopian tube samples from five healthy women. Although L1 expression quantification with the chosen technique was challenging due to the repetitive nature of the element, we found evidence of L1 expression primarily in cancer cells, but also in other cell types, e.g. cancer-associated fibroblasts. The expression levels were similar in samples taken before and after neoadjuvant chemotherapy, indicating that L1 transcriptional activity was unaffected by clinical platinum-taxane treatment. Furthermore, L1 activity was negatively associated with the expression of *MYC* target genes, a finding that supports earlier literature of *MYC* being an L1 suppressor.

## Introduction

Transposons, also referred to as “jumping genes”, are DNA elements that can change their location within a genome. They were first discovered in maize by Barbara McClintock in the 1950s^1^, but since then, they have been found in nearly all eukaryotic genomes sequenced^2^. In total, transposons make up around even 90 percent of the genome of certain plants^3^, and 50 percent of the human genome^4^. However, currently only one type of transposon, namely long interspersed nuclear element-1 (LINE-1, or L1 for short) is still capable of moving independently within the human genome, thanks to its self-encoded transposition machinery^5^. Furthermore, L1 is the most successful transposon in terms of nucleotide content, covering at least 17% of the human genome^4^.

L1s are repressed in most normal adult tissues^6,7^. However, recent studies have shown L1 activity to also occur in normal epithelia^8,9^. Nevertheless, L1 activity is significantly higher in many cancers, especially in those of epithelial origin^6,8,9^. One of these cancer types is ovarian cancer^10–12^.

L1 activity can be investigated by assessing different stages of the L1 element’s life cycle, for instance, by studying new L1 insertions, mRNA or ORF1 protein expression. To date, most studies of L1 transcription are based on bulk RNA-seq on tissue samples or cell lines^12,13^; a few single-cell level protocols have only just recently been applied^9,14,15^. Therefore, a standard workflow for this type of analysis is lacking. From a biological standpoint, it is currently unclear whether L1 expression detected in tumor samples originates purely from cancer cells, or whether the microenvironment of the tumor also shows L1 activity.

In this study, we aimed to identify L1 expression at the locus-specific level, based on 3′ single-cell RNA-sequencing (scRNA-seq), and to further compare this to L1 expression detected in bulk RNA. Our data consisted of pre- and post-neoadjuvant chemotherapy high-grade serous ovarian carcinoma (HGSOC) sample pairs from 11 patients^16^, as well as 5 normal tissue samples from healthy individuals. In addition to assessing L1 activity in different cell types, we examined whether L1 activity was associated with any changes in gene expression, and whether L1 expression changes upon chemotherapy.

## Results

### L1 expression detected in scRNA-seq data was mild

L1s are repetitive elements, meaning that aligning reads unambiguously to individual loci is far from trivial^17^. To overcome this hurdle, we quantified L1 expression based on the number of scRNA-seq reads mapping to the 1 kb downstream windows of individual L1s. This was feasible, because L1 transcription originating from the element’s own promoter occasionally extends beyond the L1 body^18,19^. Accordingly, we explored the read-through transcription from 5432 full-length L1 loci with intact promoters, as cataloged by Deininger et al.^20^, at a single-cell resolution. Out of these 5432 reference L1 loci, expression of 2432 loci were detected. However, the L1 activity was, with a few exceptions, extremely sparse. Based on unnormalized data, only 116 loci had over 100 unique molecular identifiers (UMIs) from all the samples combined. Only 9 loci had over 1000 UMIs, the maximum count being 7924 for the locus FL-L1-4738 (Supplementary Table 1, Supplementary Fig. 1A). To put these numbers into perspective, the median total UMI count for the protein-coding genes was 4430, the maximum being 11,161,580 for *FTH1* (Ferritin heavy chain 1).

Since such sparse data can be greatly skewed by non-biological factors, such as random effects from dropouts, sequencing errors, and the chosen alignment algorithm, further analysis was focused on the top 5% of the most highly active L1s only. This set of loci consisted of 124 individual L1s, all having a minimum of 95 total UMIs in the whole dataset (Supplementary Fig. 1A).

**Figure 1:**
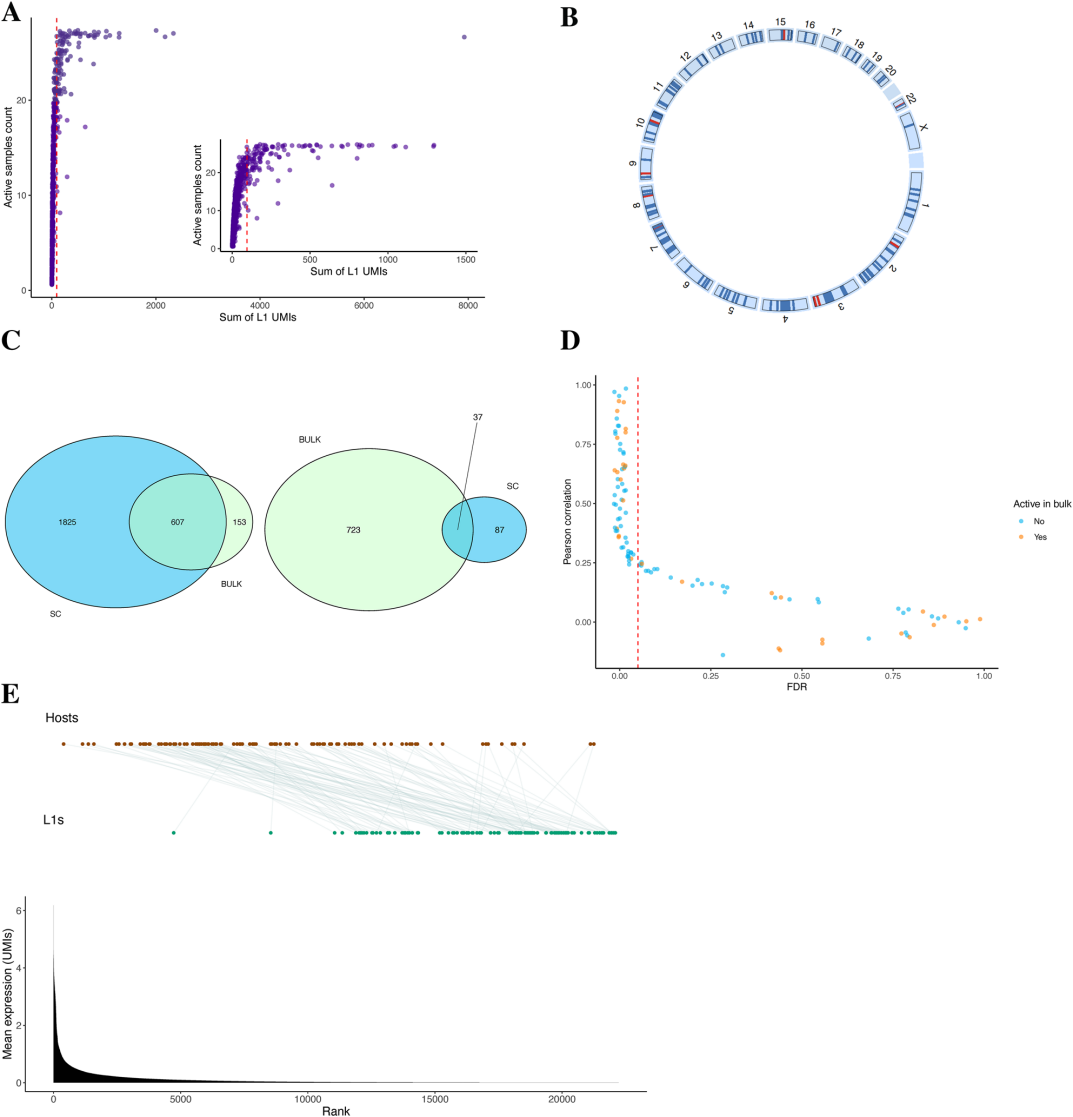
Limited overlap of transcriptionally active L1 loci detected by scRNA-seq versus bulk RNA -seq. (**A**) The relationship between total number of L1 unique molecular identifiers (UMIs) and L1-expressing samples. Each dot corresponds to a single L1 locus. Dashed red line indicates the 95th percentile of the most active L1s. (**B**) Genomic locations of the 124 most highly active L1s. L1s with a total UMI count above 1000 are indicated with red, otherwise they are colored with blue. (**C**) Overlap between total (left panel) or top 5% expressed L1 loci (right panel) detected in scRNA-seq and bulk RNA-seq. (**D**. Correlation between expression of L1s and their overlapping genes based on scRNA-seq data. Dashed red line indicates False discovery rate (FDR) = 0.05. L1s detected in both single cell and bulk RNA-seq are marked in blue, whereas scRNA-seq specific L1s are marked in orange. (**E**) Mean expression of L1s and their overlapping genes based on single cell data. The statistics are based on all samples and cell types combined. Barplot shows ranked mean expression of all genes. Green dots denote L1s, whereas brown dots denote L1-overlapping genes, ordered by rank.

Even though the majority of L1s detected had an extremely low UMI count, most were active in multiple samples (69.4%; 1689 L1 loci). The relationship between total UMI count and the number of L1-expressing samples was not linear, but instead L1s with even fewer than 100 UMIs were sometimes detected in all 27 samples (Fig. 1A). Meanwhile, there was large variation in the number of samples that expressed the top 5% L1 loci, ranging from 8 to 27 samples. The top 5% expressed L1 loci were dispersed across the genome without clear locational patterns (Fig. 1B).

### Only partial overlap of active L1s detected in bulk and scRNA-seq

In addition to tag-based 3′ scRNA-seq, we quantified L1 expression using traditional whole-transcript paired-end bulk RNA-sequencing data from the matching tumor samples. We analyzed such bulk-RNA-seq data from 18 of the tissue samples that have been previously published^16,21^, or were generated as a part of a separate study (https://www.deciderproject.eu/) and deposited in the European Genome archive under accession number EGAS00001004714. Similar to the scRNA-seq analysis, L1 quantification from bulk data was also based on the 1-kb downstream window of L1s. However, to be assigned active, a L1 locus had to have at least five times more reads on the downstream window compared to the equally sized upstream window. This was to minimize the number of false-positive loci caused by the readthrough transcription from the upstream promoters of overlapping genes. It was not possible to apply the same filtering step to the scRNA-seq data due to the 3′ nature of the reads (see “Materials and methods” for details).

When the L1s active in bulk and scRNA-seq were compared to each other, there was high overlap if all scRNA-seq-detected loci were taken into consideration; 79.9% (607) of the L1s detected in bulk RNA-seq were also detected in scRNA-seq (Fig. 1C, left panel). However, most of these loci detected with both approaches were expressed at very low level in scRNA-seq; only 29.8% (37) of the top 5% L1s of the single-cell data were detected in bulk (Fig. 1C, right panel). In addition, the majority (75.0%) of L1s detected in scRNA-seq were not detected in bulk RNA-seq (Fig. 1C, left panel).

The number of samples expressing a particular L1 varied substantially between bulk and scRNA-seq data (Supplementary Fig. 1B). Most loci detected in a high number of samples in single-cell data were detected in only a few samples in bulk. The same also applied the other way around; even though all 10 of the most highly expressed L1s from bulk RNA-seq were detected in scRNA-seq, only 4 of them were in the top 5% of the single-cell data. In other words, 6 out of 10 of these loci had less than 95 total UMIs in the entire scRNA-seq dataset.

The observed discrepancies underscore the inherent constraints of tumor sampling, and of the sequencing methodologies employed. First, the specimens analysed in bulk and scRNA-seq—while taken from the same anatomical site from the same patient—are by necessity different and therefore will to some extent vary in their tumor cell content due to intratumoral heterogeneity. Furthermore, bulk RNA-seq can detect elements that are sparsely expressed across majority of cells within a tumor, and it is not sensitive to the position of the element within the mRNA molecule, capturing a cumulative signal. The applied scRNA-seq method might overlook this signal, as it has shallower depth, and the most common method—also applied here—targets a limited 3′ region close to a polyadenylation site. Conversely, scRNA-seq can identify elements that are suitably positioned within the transcript and show pronounced expression within a subset of cells, a resolution that permits the elucidation of cellular heterogeneity and identification of unique transcriptional profiles that bulk RNA-seq might miss.

### Expression of most intragenic L1s was positively associated with expression of host genes

Since we quantified L1 expression based on the reads mapping to the L1 downstream regions instead of the L1 bodies themselves, it was expected that a large proportion of the recorded expression would originate from other transcriptionally active elements overlapping with L1s, hereafter referred to as host genes. Therefore, the relationship between expression of L1s and that of their host genes was examined. Out of all L1 loci screened, 48.5% resided in genes. Out of all the L1s detected in scRNA-seq, 58.6% were located within host genes, and this proportion grew to 88.7% in the top 5% of the most strongly expressed L1s. In other words, strongly expressed L1s were preferentially intronic, nearly nine out of ten of the loci being located within genes in the top 5% L1 group.

Focusing on the top 5% highest-expressed L1s in scRNA-seq, 58.2% of intronic loci were positively associated with expression of their host genes (FDR-adjusted p < 0.05), when Pearson’s *r* was calculated based on the sample-wise mean expression levels per cell type. Same-stranded L1-host gene pairs were more prone to having a strong correlation than opposite-stranded pairs, but both still frequently showed statistically significant association (Fig. 1D and Supplementary Fig. 2). The correlations varied between 0.99 and − 0.14, of which top seven L1s with the strongest positive correlation were same-stranded with their hosts. No L1-host pairs with negative correlation were statistically significant (FDR-adjusted p > 0.05).

Furthermore, based on scRNA-seq data, we investigated whether expression of host genes was enriched in cells that expressed the corresponding inhabitant L1, compared to cells that did not express the L1, using logistic regression models. Single-cell level association of L1 and host expression was not studied with Pearson’s correlation; since a L1 locus usually had either one or zero UMIs per cell, it was not reasonable to test for linear relationship (Supplementary Fig. 3A).When analyzing each cell type separately, the activity of 55.5% of L1s was associated with stronger host gene expression in at least one of the studied cell types (FDR-adjusted p < 0.05, Supplementary Fig. 3B,C). Of these loci, 73.8% were also positively correlated at the cell type and sample specific mean expression level (See previous section). Again, there was no statistically significant negative association between L1 and host expression.

For bulk RNA-seq data, false-positive L1s were controlled for by excluding those L1s that did not have at least five times more reads mapping to the 1 kb downstream window, compared to the reads mapping to the 1 kb upstream window. Nevertheless, those L1s that were detected both in scRNA-seq and bulk RNA-seq also frequently showed positive association with their host genes based on scRNA-seq data. Out of the 37 loci detected in both bulk and scRNA-seq, 17 showed positive correlation with their host gene at the sample level (FDR-adjusted p < 0.05), and 20 L1s showed positive association with their hosts based on the single-cell level logistic regression models (FDR-adjusted p < 0.05). These results indicate that false-positive calls of active L1 loci may remain a challenge in bulk RNA-seq despite controlling for host gene expression.

When visually investigating the relationship between mean expression of L1s and that of their host genes, the general trend was that L1s were less active (Fig. 1E). Mean expression of host genes varied substantially, with some host genes being transcriptionally inactive based on our data. Thus, transcriptionally active L1s appear to reside within both strongly and weakly expressed transcribable elements.

### L1 expression was detected in all the studied sample and cell types

**Figure 2:**
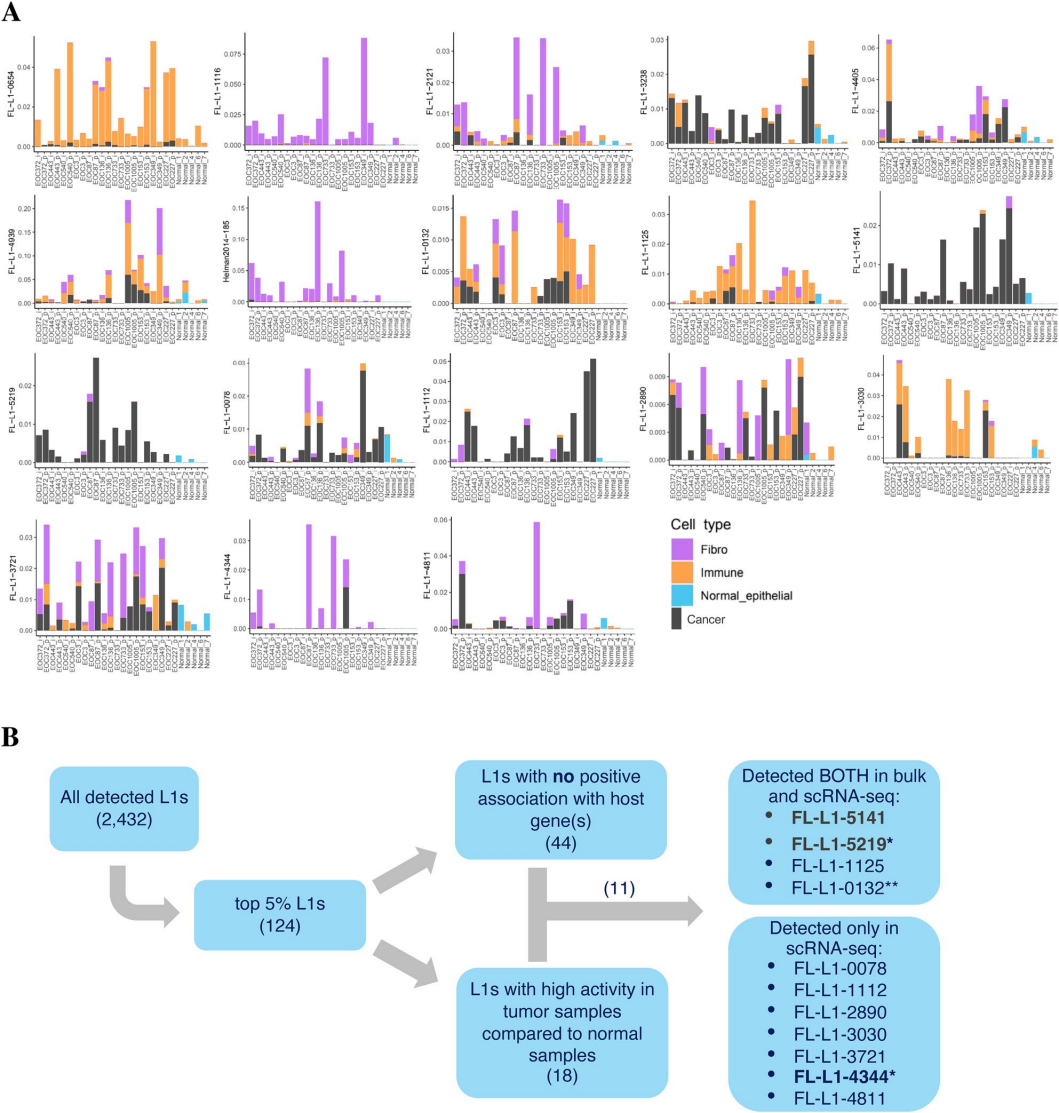
Cell type specificity of tumor sample enriched L1 loci and workflow for selection of loci to validation. (**A**) Mean expression profiles of L1s highly expressed in tumor samples compared to normal samples. Cutoff was a minimum of threefold expression in tumor samples. *EOC* Epithelial ovarian cancer, *Normal* Healthy fallopian tube/fimbria. Sample names on x axis; in tumor sample names, EOC numbers refer to patient codes, and ‘_p’ refers to pre-chemotherapy and ‘i_’ to interval, i.e. post-chemotherapy sample. (**B**) The workflow for selection of L1s of interest. The loci marked in bold were investigated with RT-PCR. *Retrotransposition competent, **Has an intact ORF2p and thus can cleave the genome.

Since L1s have substantially higher activity in tumor compared to normal tissue^7,9,22^, the expression of top 5% *L1s* in tumor samples and in normal fallopian tube samples was compared based on scRNA-seq data. An L1 locus was annotated as *Tumor sample high*, if its mean expression in tumor samples was at least threefold compared to that in normal samples. The threefold enrichment was set as a threshold based on the fold-change distribution of L1 expression between normal and tumor samples (Supplementary Fig. 3D). In total, 18 L1 loci showed high expression in tumor samples (Fig. 2A). We also examined whether some loci were *normal sample high, i.e.* highly expressed in normal samples compared to tumor samples, based on the same threefold enrichment threshold. Ten L1s showed this unexpected expression pattern (Supplementary Table 2).

We further searched for high expression in a specific cell type (threefold enrichment threshold, chosen based on the fold-change distribution of L1 expression between cancer cells versus other cell types, Supplementary Fig. 3D). The results indicated that activity of L1 loci originated from cancer cells, as well as from other cell types (fibroblasts and immune cells) among the *tumor sample high* L1s. Out of the 18 *tumor sample high* L1s, four loci were *cancer cell high* (such as L1-5141 and L1-5219), four were *fibroblast high* (such as L1-4344), and two were *immune cell high*. The remaining loci displayed a mixed expression pattern, with expression from at least two, and in many cases all three, different cell types in most samples. Interestingly, those *tumor sample high* L1 loci that were also detected in normal samples, were most often expressed in epithelial cells (Fig. 2A), especially for those L1 loci that in tumor samples showed cancer cell specific expression. In immune cells of normal samples, L1 expression was occasionally seen, and preferentially for loci that also displayed expression in immune cells of tumor samples. *Tumor sample high* L1 expression was rarely seen in fibroblasts from normal samples. All L1s showing high activity in a specific sample group or a cell type are listed and described in Supplementary Table 2.

Based on earlier studies and established properties of L1s, we further focused on the loci that (A) were unassociated with overlapping host genes and (B) were strongly expressed in tumor samples compared to normal ones^22,23^, i.e. *tumor sample high*. Out of 124 loci, 11 passed these requirements. Four loci were detected in both bulk and scRNA-seq, and seven loci were single-cell data -specific (Fig. 2B). Here, FL-L1-5219 and FL-L1-4344 are retrotransposition competent. FL-L1-0132 has an intact ORF2 but a mutated ORF1, meaning that it cannot retrotranspose but can still facilitate retrotransposition of Alu elements^24^. A summary of the studied features of all the top 5% L1s of the scRNA-seq data is provided in Supplementary Table 2.

### RT-PCR validation

To investigate whether L1 loci that showed transcriptional activity by scRNA-seq and/or bulk RNA-seq were genuinely expressed in HGSOC samples, we performed reverse transcriptase polymerase chain reaction (RT-PCR) for validation. Fresh-frozen tumor pieces were available for RNA extraction from all tumor specimens studied using scRNA-seq (Fig. 2A**),** except for EOC443_p and EOC443_i (p = pre-chemo; i = interval, post-chemo). The RT-PCR validation experiment was run for loci L1-5141, L1-5219 and L1-4344. Of these, L1-5141 and L1-5219 were active in both bulk RNA-seq and in scRNA-seq, and L1-4344 in scRNA-seq only (Fig. 2B). For each specimen, RT-PCRs were run in triplicates. It should be noted that compared to scRNA-seq and bulk RNA-seq, RT-PCR is substantially more sensitive and thus was expected to reveal more cases of locus-specific L1 expression than RNA-seq methods.

**Figure 3:**
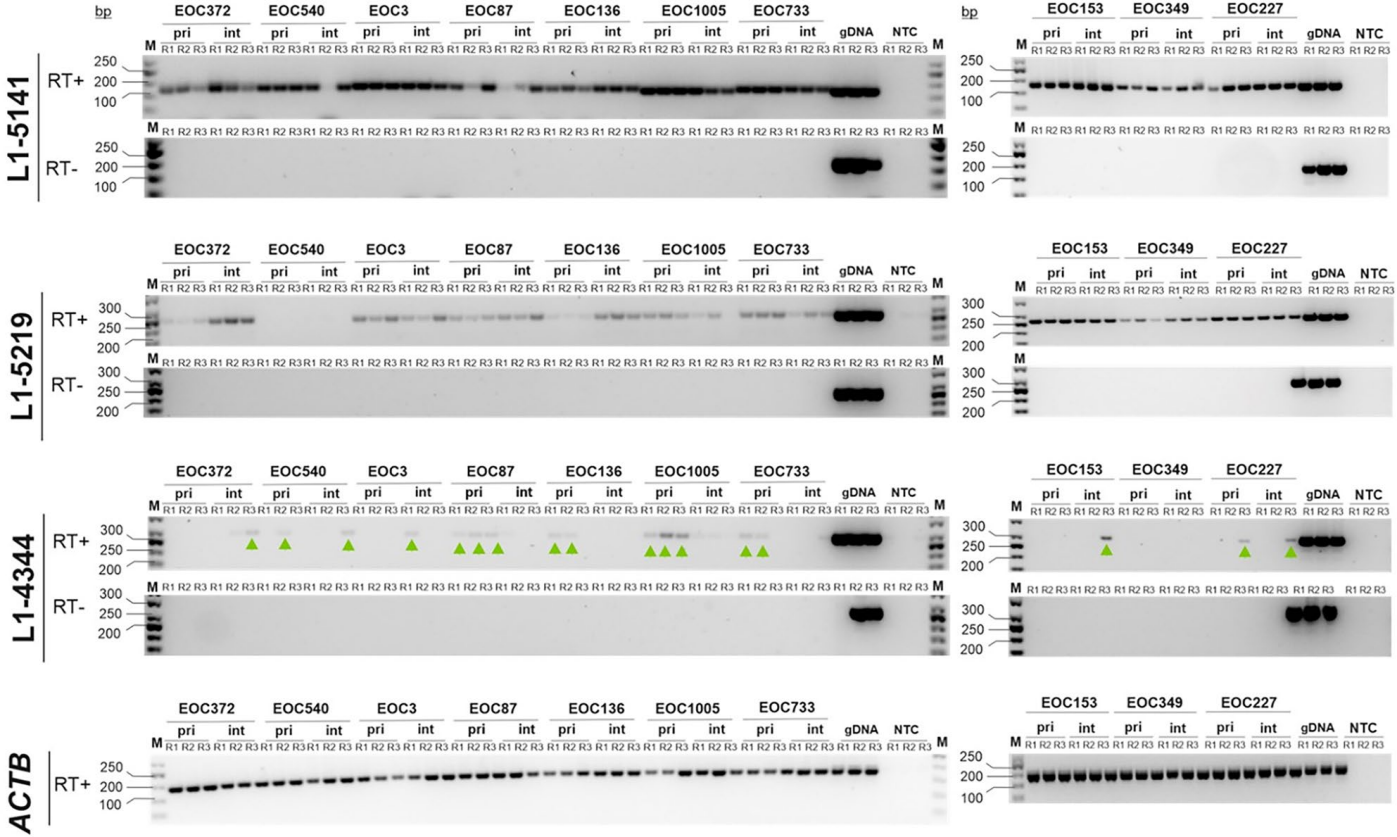
Locus-specific RT-PCR detects expression of L1-5141, L1-5219 and L1-4344. Agarose gel image of L1 locus-specific RT-PCR products, assessing expression of L1-5141 and L1-5219 (detected from both bulk- and single-cell RNA-seq analysis) and L1-4344 (detected from single-cell RNA-seq analysis only). Black and white colors of the original images were inverted for ease of visualizing fainter amplification products. The presence of L1-4344 RT-PCR products (sized 291 bp) is indicated by green arrowheads. RT-PCR with primers against ACTB transcript reports on the integrity of the RNA samples. *R1*, *R2* & *R3* replicate 1, 2 & 3 respectively; *p* primary (pre-chemotherapy); *i* interval (post-chemotherapy); *gDNA* genomic DNA; *NTC* no template control; *M* GeneRuler 50 bp DNA ladder, Thermo Scientific).

RT-PCR results for L1-5141 aligned well with scRNA-seq and bulk RNA-seq (Fig. 2) results, with all assayed tumor samples showing expression (Fig. 3, uncropped gel images available in Supplementary Fig. 4A). Pre-chemotherapy tumor sample from patient EOC136 showed L1-5141 expression by RT-PCR, although no expression of L1-5141 was detected by sc-RNA seq.

RT-PCRs for L1-5219 were also in line with RNA-seq (Fig. 2) results. As expected based on scRNA-seq and bulk RNA-seq data, most samples were positive for L1-5219 expression by RT-PCR as well. Pre- and post-chemotherapy tumor samples from patient EOC540, where no expression of L1-5219 was detected by neither scRNA-seq (Fig. 2) nor bulk RNA-seq analysis, were negative for L1-5219 expression also by RT-PCR (Fig. 3). Pre-chemotherapy sample of EOC136 (EOC136_p) was also negative for L1-5219 expression by both scRNA-seq and RT-PCR. Two samples (EOC153_i and EOC349_p) were positive for L1-5219 expression by RT-PCR (Fig. 3), despite being negative by scRNA-seq.

Of the three RT-PCR validated L1 loci, L1-4344 was distinct from L1-5141 and L1-5219 in its cell type specific expression – according to scRNA-seq data, it was predominantly expressed in fibroblasts (Fig. 2A). For L1-4344 RT-PCRs, amplification products overall were fainter than for L1-5141 and L1-5219 (Fig. 3). Moreover, for most samples, an amplification product was seen in two or less of the triplicate reactions. Taken together, RT-PCR results indicate that L1-4344 transcripts were substantially less abundant compared to L1-5141 and L1-5219 transcripts.

The only sample with robust L1-4344 cancer cell expression by scRNA-seq, EOC1005_p (a pre-chemotherapy tumor sample, Fig. 2), was the only one with 3/3 clearly positive L1-4344 RT-PCRs (Fig. 3). The two samples (EOC87_p and in EOC733_p) where scRNA-seq showed high L1-4344 expression originating from fibroblasts, were each positive in two out of three RT-PCRs. Thus, L1-4344 RT-PCR results support the notion that the fibroblast-specific expression detected by scRNA-seq in these samples is genuine. The same is true for sample EOC136_p which showed modest L1-4344 expression by scRNA-seq. In sample EOC372_p, likewise with modest L1-4344 expression by scRNA-seq, no L1-4344 transcripts were detected by RT-PCR. Several other samples were positive for L1-4344 in just one out of three RT-PCRs.

We further wanted to assess to what extent L1-4344 was expressed in a larger HGSOC patient population. For this purpose, RT-PCR was performed in an independent cohort, consisting of 35 samples (all pre-chemotherapy) from 22 patients (Supplementary Fig. 5, uncropped gel images available in Supplementary Fig. 4B). For ten patients of this cohort, multiple tumor samples from different anatomical sites were obtained, allowing us to assess intra-patient variation in L1-4344 expression. Interestingly, we identified several cases of robust L1-4344 expression in one anatomical site, but not the other(s) within the same cancer patient (e.g. patient EOC620, Supplementary Fig. 5). These results indicate intra-patient heterogeneity in locus-specific L1 transcriptional de-repression.

For tumor samples in this larger cohort, scRNA-seq data is lacking, and thus we do not know whether L1-4344 expression arises from cancer cells, from fibroblasts or from both. We note, however, that the frequency of samples expressing L1-4344 is similar to that in the scRNA-seq cohort, where L1-4344 expression primarily originated from fibroblasts. These RT-PCR results support the notion that L1-4344 expression in tumor samples, detected originally only by scRNA-seq, is genuine and relatively common.

In summary, RT-PCR results indicated that 3’ scRNA-seq detected genuinely active L1 loci, including one with fibroblast-specific expression (L1-4344) that was left undetected by bulk RNA-seq. Because L1 transcription was most robustly observed in cancer cells (by scRNA-seq and subsequent RT-PCR validation), we decided to focus our further analyses on cancer cells only. We ran the remaining analyses based on both (A) only L1-5141 and L1-5219, for which expression was detected in the majority of the samples both by bulk and scRNA-seq, and (B) all eleven loci with scRNA-seq-based expression (Fig. 2B).

### No systematic changes in L1 expression post-chemotherapy

**Figure 4:**
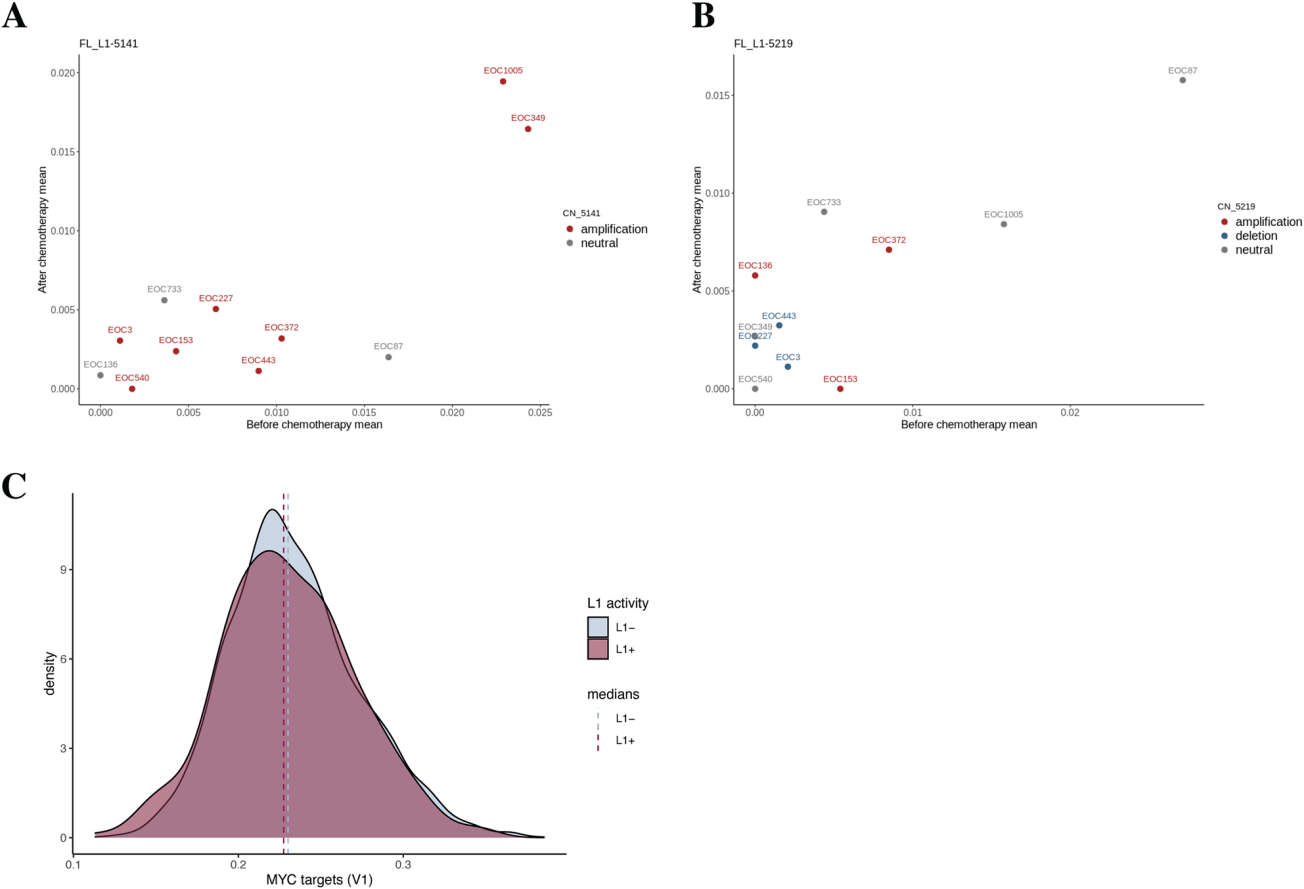
The relationship between exposure to chemotherapy and expression of the validated L1s in paired patient specimens, and pathway activity in cells by detected L1 expression. Mean expression of L1-5141 (**A**) and L1-5219 (**B**) in cancer cells of pre- and post-chemotherapy sample pairs (_p and _i, respectively). EOC codes correspond to individual epithelial ovarian cancer patients and they are colored according to the copy number status of the corresponding L1 locus region. (**C**) Area under the curve (AUC) scores for “MYC targets” hallmark gene set between L1-positive and L1-negative cells. (Bonferroni-adjusted p-value = 0.002). L1 positivity was assigned based on the expression of FL-L1-5219 and/or FL-L1-5141.

Next, we set out to assess whether the cancer cell population of paired specimens taken from the same patients before and after neoadjuvant chemotherapy expressed similar L1 patterns, and whether L1 expression systematically varies between pre- and post-chemotherapy samples. Based on scRNA-seq mean expression of 11 L1 loci, Pearson’s correlation was calculated between pre- and post-chemotherapy sample pairs. There was significant correlation for four L1 loci, including L1-5141 and L1-5219 for which there was remarkable variation between patients (Table 1, Fig. 4A,B). These two loci were located within regions that were amplified in 8 or 3 patients for L1-5141 and L1-5219, respectively, or deleted in the case of L1-5219 in 3 patients (Fig. 4A,B; colors denote the copy number status of the region). Nevertheless, the mean expression levels of these loci were not significantly different in samples with amplified or deleted against those without copy number changes in the respective region (*t* test p-value 0.59 for L1-5141; ANOVA p-value 0.44 for L1-5219). This suggests that at least within this small cohort, copy number differences do not explain inter-patient variation in expression levels of specific L1 loci.

**Table 1:** Correlation in L1 expression between pre- and post-chemotherapy sample pairs from 11 patients.

L1	Pearson’s correlation	p-value (unadjusted)	FDR-adjusted p-value
FL-L1-5141	0.81	0.003	**0.03**
FL-L1-5219	0.83	0.001	**0.02**
FL-L1-1125	0.89	< 0.001	**0.003**
FL-L1-0132	0.65	0.03	0.33
FL-L1-0078	0.02	0.96	1.00
FL-L1-1112	0.80	0.003	**0.04**
FL-L1-2890	0.24	0.48	1.00
FL-L1-3030	0.65	0.03	0.32
FL-L1-3721	− 0.13	0.70	1.00
FL-L1-4344	− 0.11	0.76	1.00
FL-L1-4811	0.42	0.20	1.00

Significant values are in bold.

*FDR* false discovery rate.

L1 expression in paired pre- and post-chemotherapy samples was compared both at the mean expression level using Wilcoxon signed-rank test, as well as at the single-cell-level expression using logistic regression models. Neither of the analyses yielded statistically significant results (p-value before multiple testing correction > 0.05). Therefore, based on our data, there is no systematic shift in L1 expression after chemotherapy exposure.

### MYC target genes were negatively associated with L1 expression

To investigate whether L1 transcriptional activity is associated with the expression of specific genes, differential expression analysis was run based on logistic regression, inferring differences in gene expression between L1 + and L1– cancer cells. As there were much less L1 + cells, and they had, on average, significantly more UMIs than the L1- cells did (Supplementary Fig. 6A,B), L1- cells were sampled and the medians of the two groups were matched. When the L1 activity cell annotations were based solely on the RT-PCR validated, *cancer cell high* loci L1-5141 and L1-5219, only the expression of *FAU* was statistically significantly different between the studied groups, being slightly more active in L1- cells (Bonferroni-adjusted p = 0.003, average logFC = − 0.07). There were no significant cell cycle differences between these L1 + and L1 cells in our data set (172/2311 cells in G1, 73/1077 cells in S and 39/490 cells in G2/M in L1 + /L1– cells, respectively; chi-squared test p-value 0.70).

When the differential gene expression analysis was repeated on cells annotated L1 + /L1– based on the detection of any of the L1 loci that appeared to be active based on scRNA-seq (the eleven loci shown in Fig. 2B), there were two genes slightly more active in L1- cells, namely, *PRDX1* (Bonferroni-adjusted p = 0.001, average logFC = − 0.06) and *TMEM126A* (Bonferroni-adjusted p = 0.007, average logFC = − 0.15). *FAU* was again enriched in L1– cells, but the difference was not statistically significant after the multiple testing correction (Bonferroni-adjusted p = 0.40, average logFC = − 0.07).

As another approach, differential expression analysis was repeated at the gene set level. First, AUCell^25^ was utilized for quantifying the activity of gene sets from Molecular signatures database^26^. AUCell is based on area under the curve, with which the enrichment of genes participating in a particular gene set can be inferred. The obtained AUC score matrix was then used as an input for differential gene set expression analysis, which was executed similarly as above for individual genes. When the L1 activity annotations were based solely on the loci L1-5141 and L1-5219, the only statistically significant finding was MYC targets (V1 = variant 1), consisting of 200 genes. The gene set was found to be more active in L1- cells (Bonferroni-adjusted p = 0.002 average logFC = -0.005) (Fig. 4C). MYC targets V2 gene set (58 independent genes) was not statistically significantly differentially expressed. When the L1 + /L1– annotations were based on the 11 scRNA-seq detected loci shown in Fig. 2B, MYC targets (V1) were again downregulated in the L1 + cells (Bonferroni-adjusted p < 0.001, average logFC = − 0.006 ), along with the reactive oxygen species pathway (Bonferroni-adjusted p = 0.008, average logFC = − 0.008; Supplementary Fig. 6C and D, respectively.).

To ensure that the obtained findings were not arbitrary, the same analyses were run using random L1 + and L1– cancer cell annotations. The number of L1 + cells was matched to that of earlier analysis. No statistically significant findings were found either at the single gene or gene set level.

## Discussion

Upregulated L1 activity is a well-known feature of many epithelial cancer types^27^, including ovarian carcinoma^10–12^. However, single-cell level analysis of L1 expression has been lacking for HGSOC. Hereby, our study aimed to examine locus-specific L1 expression in both pre- and post-chemotherapy HGSOC specimens, as well as normal fallopian tube samples using scRNA-seq data. The detected activity was low, and global L1 expression was spread across all cell types. The 3′ scRNA-seq data of L1 expression was sparse and potentially included many false-positive loci. Nevertheless, we identified eleven L1 loci that were expressed especially in tumor specimens, and further lacked correlation with their host genes. The implication of these active loci on tumors are varied: (1) Two out of these eleven loci are capable of retrotransposition, allowing them to copy and reintegrate into the genome, potentially causing instability; (2) One locus has a functional ORF2 region and thus can cause double-strand breaks in the host genome^28^ and/or also facilitate mobilization of Alu elements^29^; and (3) The remaining eight loci are full-length L1s with intact promoters. While they are not retrotransposition-competent, their expression could disrupt the transcriptional landscape around them, possibly influencing cancer progression through various local effects. The activity of some of these L1s were strongly concentrated on cancer cells, but other L1s were found to be expressed at higher levels in other cell types within the tumor samples, i.e., immune cells or fibroblasts.

Fibroblasts in healthy fallopian tube samples showed negligible L1 activity, in agreement with previous studies of fibroblasts in the normal epithelium^8,9^. Our finding that fibroblasts in tumor samples showed signal in certain L1 loci – validated by RT-PCR for L1-4344 – was thus surprising. To our knowledge, this constitutes the first report of likely L1 expression in cancer-associated fibroblasts (CAFs). CAF-specific L1 activity likely reflects the epigenetic dysregulation of fibroblasts residing in the tumor microenvironment. It is also important to note that L1 retrotransposition events has been shown to positively correlate with age^8,9^, and the average age of the HGSOC cohort analysed was 67 years.

Furthermore, L1 transcriptional activity was not restricted to tumor samples only – many of the loci showed distinct activity in healthy samples as well. This was also true for many cases detected both in bulk and scRNA-seq. Even though this finding may be caused by the presence of false-positive loci, emerging evidence indicates that unlike previously thought, L1 activity in certain tissues is not restricted to malignant cells^8,9^, and our findings suggest that this is true for normal fallopian tube epithelium as well.

There was a strong bias towards the activity of intronic L1s compared to intergenic ones. Furthermore, the expression of the majority of intronic L1s was statistically significantly positively associated with that of their host genes. L1 expression results from both bulk and scRNA-seq may therefore include many false-positive active loci; to mitigate this, we focused on L1 loci that lacked positive association with their host genes. In conclusion, our results once again demonstrate the previously reported challenges of transposon expression quantification^17^, and highlight the importance of controlling for them.

At the same time, it is important to acknowledge that transcriptional activity of L1s was likely underestimated in our study. First, scRNA-seq data is known for being shallow, as on average, only 14–15% of the mRNA per cell is sequenced with the utilized reagent kit^30^. Second, since our approach was to only quantify those L1 transcripts that elongated beyond the polyadenylation site of the L1 element, transcripts that terminated normally were not detected. Lastly, the downstream window next to an L1 body was set to 1 kb, meaning that those L1s that potentially transcribed further downstream were also excluded from the analysis. Tubio et al. reported that the L1 transduction events in cancer were usually less than 1 kb, but the longest copy had around 12 kb of downstream sequence^31^. The length of L1 downstream sequence in the mRNA can therefore elongate well behind the chosen 1-kb limit. These defects lead to our data likely containing a significant amount of false-negative calls, which reduces the power of the performed analysis.

However, we were unable to estimate the actual level of false positive or false negative calls due to the lack of ground truth of single-cell locus-specific L1 expression in clinical specimens, and hence the specificity or sensitivity of the approach, respectively, remain unknown.

With our single-cell level L1 expression identification approach, it is possible to yield locus-specific results. It is an advantageous feature, as it enables, for example, studying the association between L1s and their overlapping or neighboring genes. Some previous studies have quantified L1 expression by using collapsed “metagenes”^14^ or a single consensus L1 promoter^9^. With these approaches, it is possible to align multimapping reads, and therefore to obtain more comprehensive expression quantification data. Some single-cell level studies before us have also aimed to quantify L1 expression at the individual locus level, but have stumbled upon similar issues in frequent false-positive L1s and very low overall expression^15^. All in all, the current L1 expression quantification methods utilizing high-throughput and cost-effective scRNA-seq appear to require a choice between one or the other – either having an abundance of spurious L1 expression or being incapable to detect the expression of individual loci. The choice must be made based on the needs of the research project in question.

Coming back to our own results, expression of none of the assayed L1 loci significantly varied between pre- and post-chemotherapy sample groups. In contrast, a study by Guler et al. performed in a homogenous ex vivo lung cancer model, found that the overall L1 expression increased due to carboplatin treatment^32^. However, the subpopulation of drug-tolerant cells remaining after the treatment showed repressed L1 expression due to the increase in repressive histone marks. Another study showed that L1s were activated in mouse hematopoietic stem cells in response to chemotherapy^33^. It is possible that since our analysis was only based on a few individual loci, along with the fact that our data was enriched with false zeroes, it was not achievable to find statistically significant differences in expression between pre- and post-chemotherapy samples. On the other hand, L1 upregulation in response to chemotherapy may only occur ex vivo, outside of the context of heterogeneous tissue in vivo. Consequently, the real-world clinical samples utilized in our study have potential to paint a more physiologically relevant picture of this phenomenon. The question should therefore be further examined with complementary methods, such as RNA in situ hybridization, in the future.

By investigating differential expression at the molecular signature gene set level between L1 + and L1- cells, the expression of MYC target genes was found to be higher in L1- cells. MYC has been previously reported as a likely L1 repressor, as *MYC* expression negatively correlated with L1 in The Cancer Genome Atlas breast and ovarian tumor samples^34^. Furthermore, the same study also showed that MYC physically binds to the L1 promoter in multiple cell lines, and by post-transcriptionally knocking out MYC in HEK293 cells, L1 promoter activity increases^34^. Our findings from the differential gene set expression analysis at the single-cell level are in line with these results.

To date, it has been repeatedly shown that L1 activity can impact the function of its host cell, and that L1s become more transcriptionally active in many cancer types. Therefore, there is a clear need for further studies of L1 expression and its effects in cancer. With the advancements in this field, it could be possible to find new biomarkers for both cancer detection and prognosis, as well as to develop novel treatments. We expect to keep seeing improvements in the methods of L1 expression quantification in the future, and hope that the findings of our study can be of benefit for this process.

## Materials and methods

### Patient recruitment

All HGSOC and non-cancerous samples were collected from patients who provided written informed consent before their enrolment in the study. The Ethics Committee of the Hospital District of Southwest Finland approved the study under decision number EMTK: 145/1801/2015. All experiments were performed in accordance with the Declaration of Helsinki.

### Preparation of scRNA-seq samples

In addition to our previously published scRNA-seq samples from 11 patients collected concurrently with both laparoscopy and interval debulking surgery^16^, non-cancerous fallopian tube or fimbria tissue samples were collected from 5 healthy post-menopausal women. Immediately after surgery, the samples were incubated overnight in a mixture of collagenase and hyaluronidase (Department of Pathology, University of Turku) to obtain single cell suspensions. These were subsequently processed with the standard Chromium Single Cell 3′ Reagent Kit v. 2.0 (10 × Genomics) protocol for single cell RNA-sequencing with Illumina HiSeq4000 (Jussi Taipale Lab, Karolinska Institute, and Institute for Molecular Medicine Finland).

### Quality control and alignment of scRNA-seq data

Reads originating from poor-quality cells were excluded (quality control steps done and explained by Zhang et al.^16^). After that, STAR 2.7.8a^35^ was used to perform alignment. Genome indices were generated based on the GRCh38.d1.vd1 reference genome and GENCODE v25 annotation. To reduce dubious alignments to the L1 polyadenine stretches, a maximum of two mismatches per read was allowed. The workflow for alignment and barcode/UMI extraction (explained below) was run within the Anduril 1 framework^36^.

### Extraction of L1 reads from the scRNA-seq data

L1s often overlap with other transcriptionally active elements. Therefore, reads mapping more than 1 percent or more to exons were excluded to reduce the number of reads from gene-derived transcripts. In addition, reads mapping 90 percent or more to repeats were excluded to decrease the number of reads mapping ambiguously to repetitive regions. The annotations were derived from RepeatMasker (http://www.repeatmasker.org). The described steps were performed with Intersect from Pybedtools (v0.8.1), a wrapper of Bedtools^37,38^.

Since the L1 loci of the human genome are both remarkably numerous and highly similar to each other in sequence, their expression is difficult to quantify at the single-locus level. However, since the elements are known to have read-through transcription past their weak polyadenylation site^18^, we quantified L1 expression by counting UMIs from the 1 kb downstream windows of the individual L1 loci. To exclude L1s that are not authentically active but rather are only incorporated in the host-gene transcripts, we also quantified UMIs mapping to the 1 kb upstream windows of L1s. The number of upstream-mapping UMIs was subsequently compared with that of the downstream-mapping UMIs to ensure there being significantly more downstream-mapping UMIs (See part Evaluation of the L1 expression from the scRNA-seq data). Similar ideas have been successfully implemented in earlier studies^20,39^.

To extract reads mapping to the described windows, Intersect was again used to only retain reads with a minimum overlap of 60 percent with the reference regions. The 1 kb reference windows were based on the list from Deininger et al^20^, consisting of full-length L1 loci with intact promoters. The threshold of 60 percent was selected as optimal since it excluded aberrant splice junction reads, while preserving many reads that did not fully overlap the small and artificially set 1 kb window.

In some cases, the utilized L1 reference list was imprecise in coordinates; the polyA tail of the L1 sometimes extended to the 1 kb downstream window, which caused imprecision in the unpaired scRNA-seq alignments. To exclude reads that mapped ambiguously to polyA regions, Pysam 0.16.0.1 (https://github.com/pysam-developers/pysam), a python wrapper of SAMtools^40^ was used to exclude reads for which the A or T base composition was 60 percent or higher.

### Expression quantification from the scRNA-seq data

Based on scRNA-seq, the expression of both L1s and the rest of the genome was quantified with UMI-tools 1.1.1^41^. Before the alignment, cell barcodes and UMIs were extracted from the reads. Subsequently, the unfiltered reads were assigned straight to the genome (featureCounts from Subread v2.0.1^42^), and the filtered reads (see: Extraction of L1 reads) were assigned to the L1 1 kb upstream and downstream windows. Only reads that mapped uniquely and to the same strand as the reference were considered. The assigned reads were then deduplicated and counted per feature per cell, by counting upstream and downstream reads separately for each L1 locus.

### Evaluation of L1 expression from the scRNA-seq data

After counting the UMIS from the 1 kb downstream and upstream windows of each L1 locus per cell based on scRNA-seq data, it was soon apparent that minimal activity could be detected in the upstream windows. Out of all 2432 detected L1s, only 224 loci would be filtered out even if the downstream-upstream ratio threshold was set as high as 10:1. This finding can be explained by the utilized scRNA-seq protocol; the obtained reads were 3′ selected, meaning that only the 3′ end of the transcript fragments were sequenced. Therefore, we decided on not using the upstream read counts in the evaluation of L1 expression in the scRNA-seq data, but instead searched for alternative solutions to distinguish read-through transcription from real, L1-promoter-derived, activity.

Most of the over 2000 detected loci were extremely lowly expressed, nearly 30 percent of them having only a single UMI total from all samples combined (See Supplementary Table 1 for more information). Therefore, only the top 5% of most strongly expressed L1s were included in further analysis. Consequently, only L1s with a minimum of 95 UMIs total were kept. The same threshold was subsequently utilized to filter out very mildly expressed genes. After these initial filtering steps, the data was normalized using the NormalizeData function from Seurat v4.0.3^43^ with its default parameters.

Earlier studies have found that L1s are much more strongly active in tumors compared to normal tissue^7,9,22,44^. Therefore, we investigated whether the expression of individual L1 loci was enriched in our scRNA-seq tumor samples compared to normal samples. To achieve this, the mean expression per sample per cell type was calculated for each L1. If the mean expression of a L1 was more than threefold in tumor samples compared to that of the normal samples, the locus was considered to be *tumor sample high*. The same comparison was done also between the mean expression in cancer cells and each other cell type (cell annotations created and explained by Zhang et al.^16^). The threefold enrichment was set as a threshold based on the fold-change distributions of L1 expression between normal and tumor samples, as well as the fold-change distributions of L1 expression between tumor and other cell types (Supplementary Fig. 3D). The reason for not studying tumor sample enrichment using statistical methods was that the L1 expression detected with scRNA-seq was extremely mild, and the data was likely inflated with false-zero counts. Furthermore, the sample count, especially in the case of normal tissue, was low. Therefore, the sensitivity of statistical analysis would have been extremely poor.

In addition, we investigated the association between the expression of L1s and their host genes. The expression of L1s and hosts can be associated due to many reasons; for example, an L1 could benefit from host-expression due to open chromatin structure, making them coexpress more frequently. However, L1 and host gene could also erroneously appear to be coexpressed because the reads mapping to the downstream window of L1 in question are solely resulting from the expression of the host, as opposed to the L1 itself. Therefore, we reasoned that if there is correlation, the activity of the L1 in question may be ingenuine. The association was studied in two ways. First, based on mean expression per sample per cell type, Pearson’s correlation between a host and a L1 was calculated. Second, cell types were investigated separately at the single-cell level by generating a logistic regression model for each L1 so that the dependent variable was L1 activity (L1 + /L1–), and the independent variable was host gene expression. In addition, sample ID was used as an independent variable to adjust for sample bias. Single-cell level association was not studied with Pearson’s correlation, because L1 loci usually had only one or zero UMIs per cell. Therefore, it was not reasonable to test for linear relationship (Supplementary Fig. 3A).

Since L1 expression was nearly always bimodal (1/0 UMIs), it was also apparent that normalization was not capable of removing cell size bias from the single-cell level association analysis; cells with a larger number of UMIs were much more likely to be L1-positive than those with less UMIs (Supplementary Fig. 6A,B). Therefore, for each L1 locus studied, we matched the medians of L1-expressing and L1 non-expressing cells; the L1- cells were first ordered by their total UMI count, after which the same number of cells was selected from each side of the median UMI count of L1 + cells. This subset, along with the L1 + cells, was then used for the subsequent coexpression analysis.

Based on the analysis described above, L1s were selected as candidates for RT-PCR validation and further analysis, when they A) Were 3 times more strongly expressed in tumor samples when compared to normals and B) showed no positive association with their host genes based on either of the tests (logistic regression model and Pearson’s correlation).

### Data analysis to detect active L1 loci from bulk transcriptomic data

We utilized RNA-seq data generated from 18 HGSOC samples from previous studies^16,21^; Decider Project (https://www.deciderproject.eu/)) to assess L1 transcriptional expression. Since L1 transcripts with 3 ´ transductions can be detected with high reliability^20,39^, we aimed to detect them using the bulk RNA-seq data. Alignment files obtained in BAM file format were filtered for: (A) all the reads that mapped in exonic regions and (B) all the reads that mapped to repeat regions, by 90% using bedtools intersect^38^. Forward reads and reverse reads were then separated using samtools^40^ using flags 67 and 131, respectively. Subsequently, these forward and reverse reads were intersected with 1 kb region both upstream and downstream of all potentially transcribing L1s^20^ in a strand-aware manner. The RNA fragments mapping on both upstream and downstream regions was counted using htseq-count^45^. A L1 locus was deemed to be “expressed” if the number of RNA fragments mapped on its 1 kb downstream window was at least 5 times as much as those mapped on its 1 kb upstream window.

### Reverse-transcriptase PCR (RT-PCR) for L1 expression validation

RT-PCR was used to validate the expression of L1 loci that were detected by scRNA-seq analysis, or by both scRNA-seq and bulk RNA-seq analysis. To do this, complementary DNA (cDNA) was synthesized from 1 µg of total RNA extracted from tumor samples by SuperScriptTM IV Reverse Transcriptase (Thermo Scientific, catalog no.: 18090010) using Oligo dT primers (Thermo Scientific, catalog no.: 18418020) according to the manufacturer’s instructions. The generated cDNA was diluted in nuclease-free water at a ratio of 1:10 and amplified using a common forward primer targeting the 3´-end of L1HS^46^ and a locus-specific reverse primer (Supplementary Table 3) targeting the non-repetitive 3´ flanking region of the selected L1 locus using Phusion Hot Start II DNA polymerase (Thermo Scientific, catalog no.: F549L). 6 µl of these RT-PCR products were then run on 1% (w/v) agarose gel containing GelRed® Nucleic Acid Gel Stain, visualized, and qualitatively scored for presence or absence of L1 expression. RT-PCR was also performed on “RT-” control, which used as a template total RNA from the same sample which went through all the steps for cDNA synthesis without reverse-transcriptase (RT) enzyme. RT- controls were performed to check if the RNA was contaminated with genomic DNA. As a positive control, primers targeted at *ACTB* gene (Supplementary Table 3) were used with RT + cDNA as template; these reactions were used to assess whether RNA used for cDNA synthesis and subsequent RT-PCR was intact.

### Differences in L1 expression between pre- and post-chemotherapy scRNA-seq samples

Since L1 transcription was most robustly observed in cancer cells (by the RNA-seq analyses and subsequent RT-PCR validation), we decided to focus our further analysis on cancer cells only. For the L1s appearing to be genuinely expressed based on the scRNA-seq analysis (N = 11), we investigated the differences in expression between pre- and post-chemotherapy sample pairs. Based on mean expression levels per sample per cell type, Pearson’s correlation for the pre- and post-chemotherapy sample pairs was calculated.

To search for differences in L1 expression between pre- and post-chemotherapy samples, Wilcoxon signed-rank test was executed based on the sample means. To investigate the same question at the single cell level, a logistic regression model was created, in which the dependent variable was the pre/post-chemotherapy group membership, and the independent variables were the mean expression of a validated L1 and Patient id, the latter adjusting for bias.

### The relationship between L1 and gene expression in scRNA-seq

To study whether L1 expression is associated with specific changes in gene expression, differential gene expression analysis was run with FindMarkers from Seurat v4.0.3. using logistic regression and adjusting for sample bias (parameter latent.vars = Sample.ID). L1 expression of a cell was annotated L1 + if a cell expressed one or more L1 loci, and L1- if none of the loci were active. These annotations were done based on both A) the two L1s with most evidence of genuine activity (active in both bulk and scRNA-seq, RT-PCR-validated), as well as B) all active L1s with high tumor sample activity and without host gene coexpression, based on scRNA-seq analysis (N = 11). The genes that were expressed in a minimum of 5% of the cells were tested, and the log fold change threshold was set to 0.05 to enhance the sensitivity. High sensitivity was considered important, since many genes previously reported to be possible L1 regulators, such as those participating in DNA repair^47^, were mildly expressed in our dataset. Lastly, to ensure that the significant findings were not associated with differences in L1 host gene expression, host gene expression was used as an additional, binary (host + /host-) independent variable.

Since a large number of dropouts (i.e. false-zeroes) is typical for scRNA-seq data, it is likely that differential gene expression analysis is not a sensitive enough method to detect all expressional differences between L1 + and L1- cells. Therefore, we further investigated differential expression at the gene set level, using 50 hallmark gene sets from molecular signatures database^26^. Hallmark gene set expression scores were calculated with AUCell 1.12.0^25^, which uses the area under the curve to score enrichment in expression for each gene set per cell. Parameters of AUCell were left as default. Based on the AUC score matrix, FindMarkers was run similarly as before, but lowering the log fold-change threshold to 0.005.

Since the differential expression analyses explained above were also vulnerable to cell size bias, the UMI counts of L1 + and L1– cells were matched as before by subsampling the L1- cells (See “Evaluation of L1 expression”).

To ensure that the results obtained from the differential expression analyses were not coincidental, both the gene level and gene set level differential expression analyses were repeated with randomized L1 + and L1- annotations. The number of L1 + cells was matched to that of the analyses explained above.

To identify the copy number profiles of the L1 locus regions, InferCNV v1.7.1^48^ R package was utilized. The copy number status (amplified, neutral or deleted) was determined for individual cells, and patients were categorized into these groups based on the majority status of individual cells. To assign cell cycle phases, the CellCycleScoring function from package Seurat was applied.

### Supplementary Information

Supplementary Information.

## Data Availability

Raw scRNA-seq data are deposited in the European Genome-phenome Archive (EGA; accession code EGAS00001006246 for normal samples, EGAS00001005010 for tumor samples). L1 expression count data from all the samples, as well as gene expression count data from the tumor samples, based on scRNA-seq, are available in Gene Expression Omnibus (GEO) with accession code GSE235329.
