# Supplementary material for: Locus-specific LINE-1 expression in clinical ovarian cancer specimens at the single-cell level
Source: Sci Rep. 2024 Feb 21;14:4322. doi: 10.1038/s41598-024-54113-w (PMC10881972; doi:10.1038/s41598-024-54113-w)
Supplement: Supplementary file 1 — Supplementary Information. [file 41598_2024_54113_MOESM1_ESM.pdf]

Locus-specific LINE-1 expression in clinical ovarian cancer specimens at the single-cell level

Anna Perkiö [1], Barun Pradhan [1,a,b], Fatih Genc [1], Anna Pirttikoski [1], Sanna Pikkusaari [1], Erdogan Pekcan Erkan[1,c], Matias Marin Falco [1], Kaisa Huhtinen [1, 2], Sara Narva [3], Johanna Hynninen [3] , Liisa Kauppi [1,*] and Anna Vähärautio [1,4*]

1 Research Program in Systems Oncology, Research Programs Unit, Faculty of Medicine, University of Helsinki, Helsinki, 00290, Finland
2 Institute of Biomedicine and FICAN West Cancer Centre, University of Turku and Turku University Hospital, 20521 Turku, Finland
3 Department of Obstetrics and Gynecology, University of Turku and Turku University Hospital, 20521 Turku, Finland
4 Foundation for the Finnish Cancer Institute (FCI), Helsinki, Finland

a Current affiliation: Sandra and Edward Meyer Cancer Center, Weill Cornell Medicine, New York, NY, USA
b Current affiliation: New York Genome Center, New York, NY, USA
c Current affiliation: Faculty of Medicine and Health Technology, Tampere University, Tampere, Finland

* Correspondence: liisa.kauppi@helsinki.fi (L.K.), anna.vaharautio@helsinki.fi (A.V.)

Supplementary information

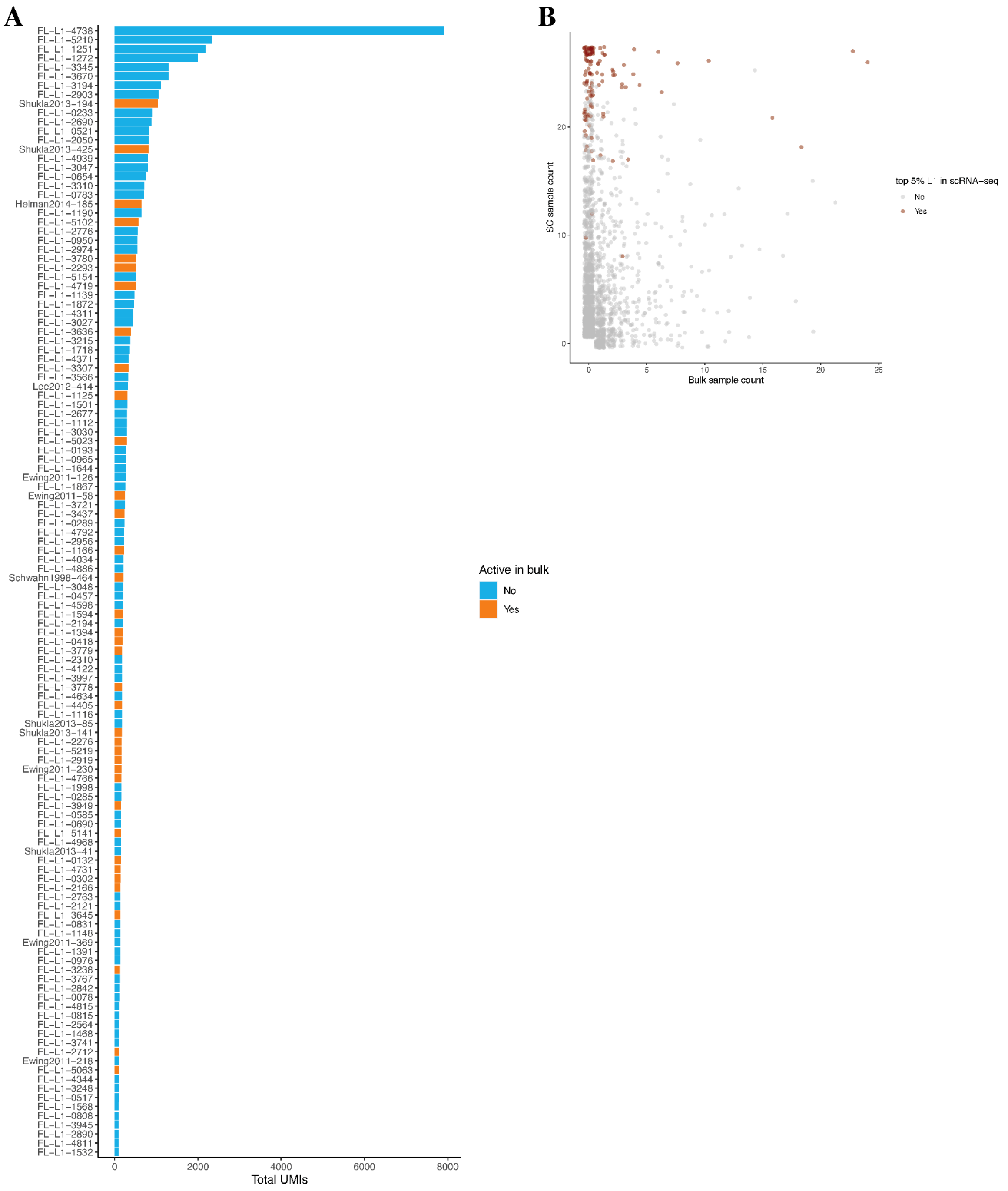

Supplementary Figure 1. A. *Total number of UMIs per L1 in unnormalized single cell dataset. L1s that were also detected in bulk RNA-seq are marked in orange.* ***B****. The number of L1-expressing samples in bulk and scRNA-seq data. One dot corresponds to a single L1 locus. The most active L1s of the scRNA-seq (top 5% based on total UMI count) are marked in blue.*

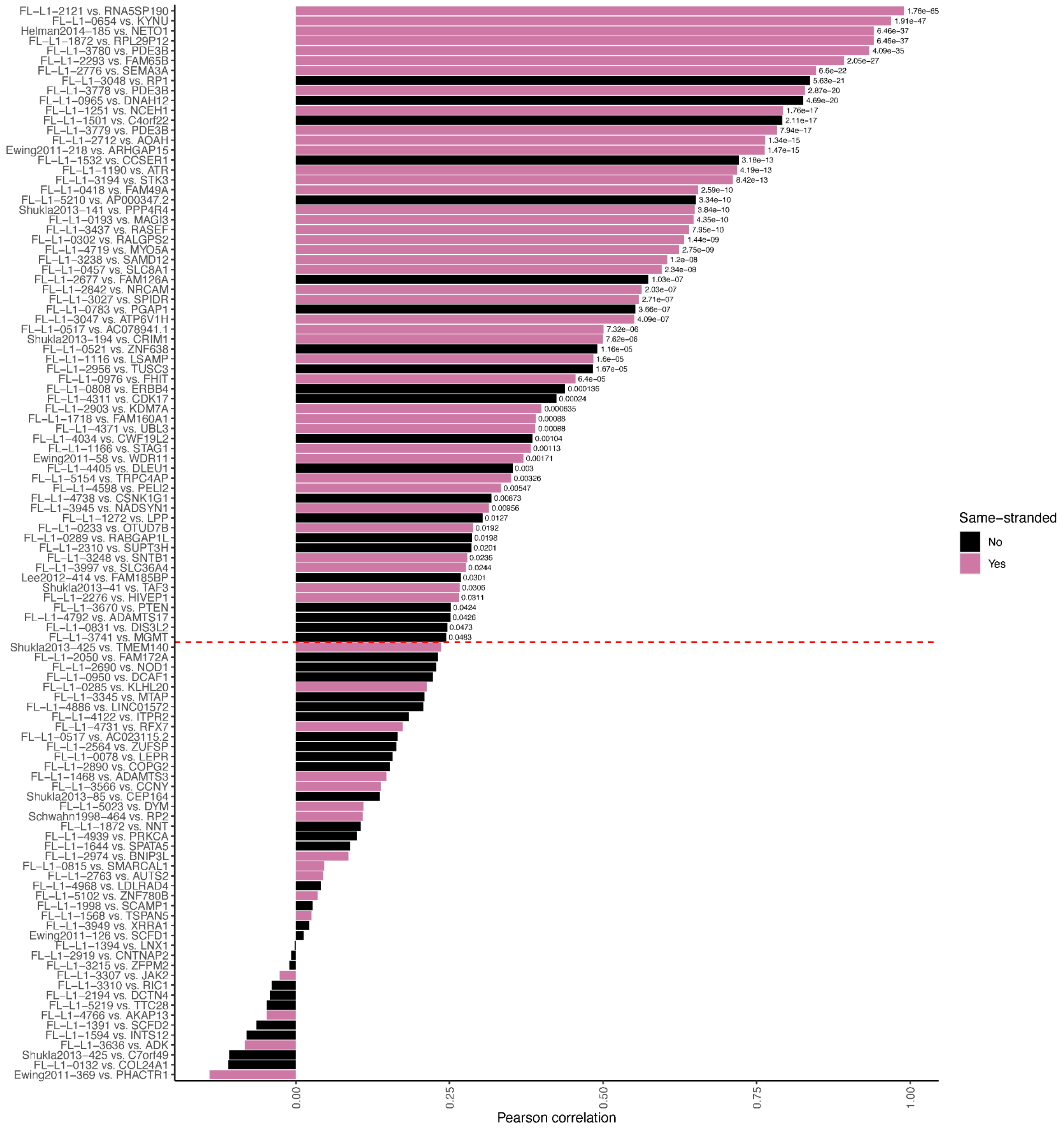

Supplementary Figure 2. *Pearson's correlation coefficients between L1s and their host genes from highest to lowest. Each bar represents the correlation coefficient between a single L1-host pair, calculated based on their mean expression per sample per cell type. Numbers next to bars indicate false discovery rate -adjusted p-values (FDR). Pairs above the dashed red line are statistically significantly associated (FDR < 0.05).*

A

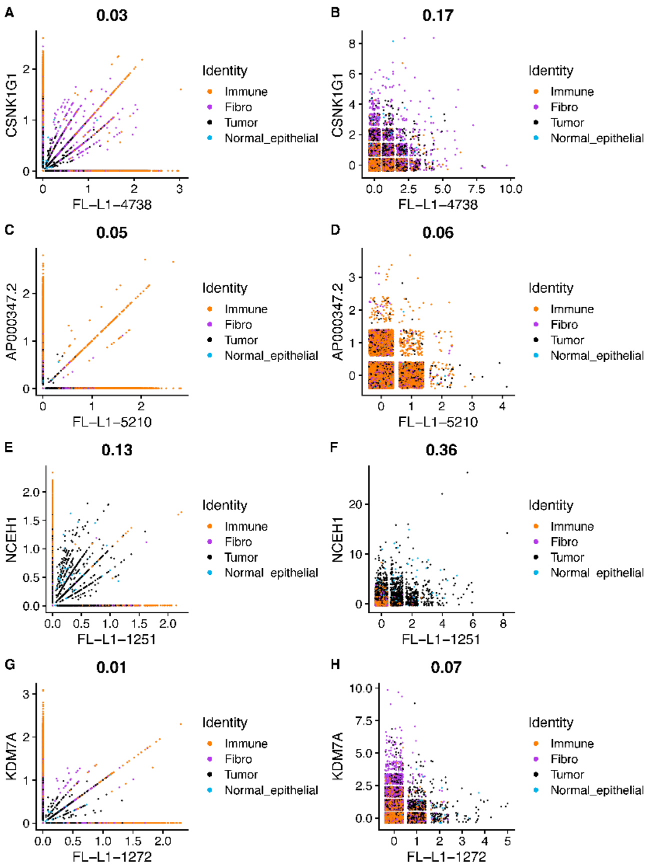

B

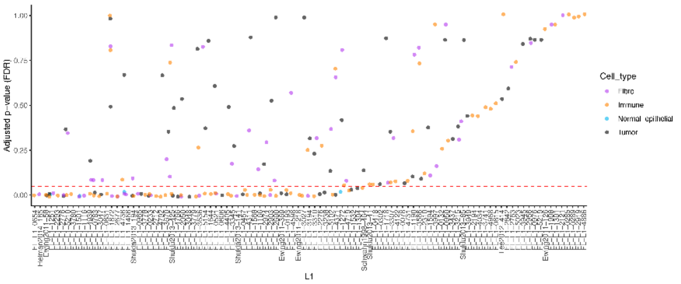

C

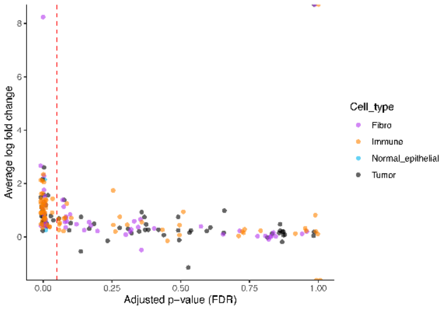

D

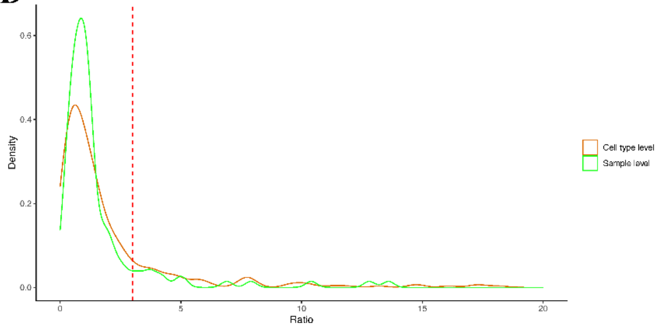

Supplementary Figure 3. A. *Expression of the four most highly active L1s of scRNA-seq data and their host genes. The graphs on the left (A,C,E,G) are based on log-normalized counts, whereas graphs on the right (B,D,F,H) are based on unnormalized counts.* ***B.*** *Adjusted p-values of host gene differential expression analysis between cells that do and do not express the resident L1 in question. Dot denotes a host gene in a particular cell type. FDR = false discovery rate.* ***C.*** *Differential expression of L1 host genes between cells that do and do not express the corresponding resident L1.*

Positive average log fold change values indicate higher host gene expression in the resident L1-expressing cells. ***D.*** *Distribution of mean expression ratios between tumor and normal samples ('Sample level'), as well as between cancer cells and other cell types ('Cell type level'). A single value corresponds to a single L1 locus. Dashed red line indicates the selected threshold for the enrichment (3:1).*

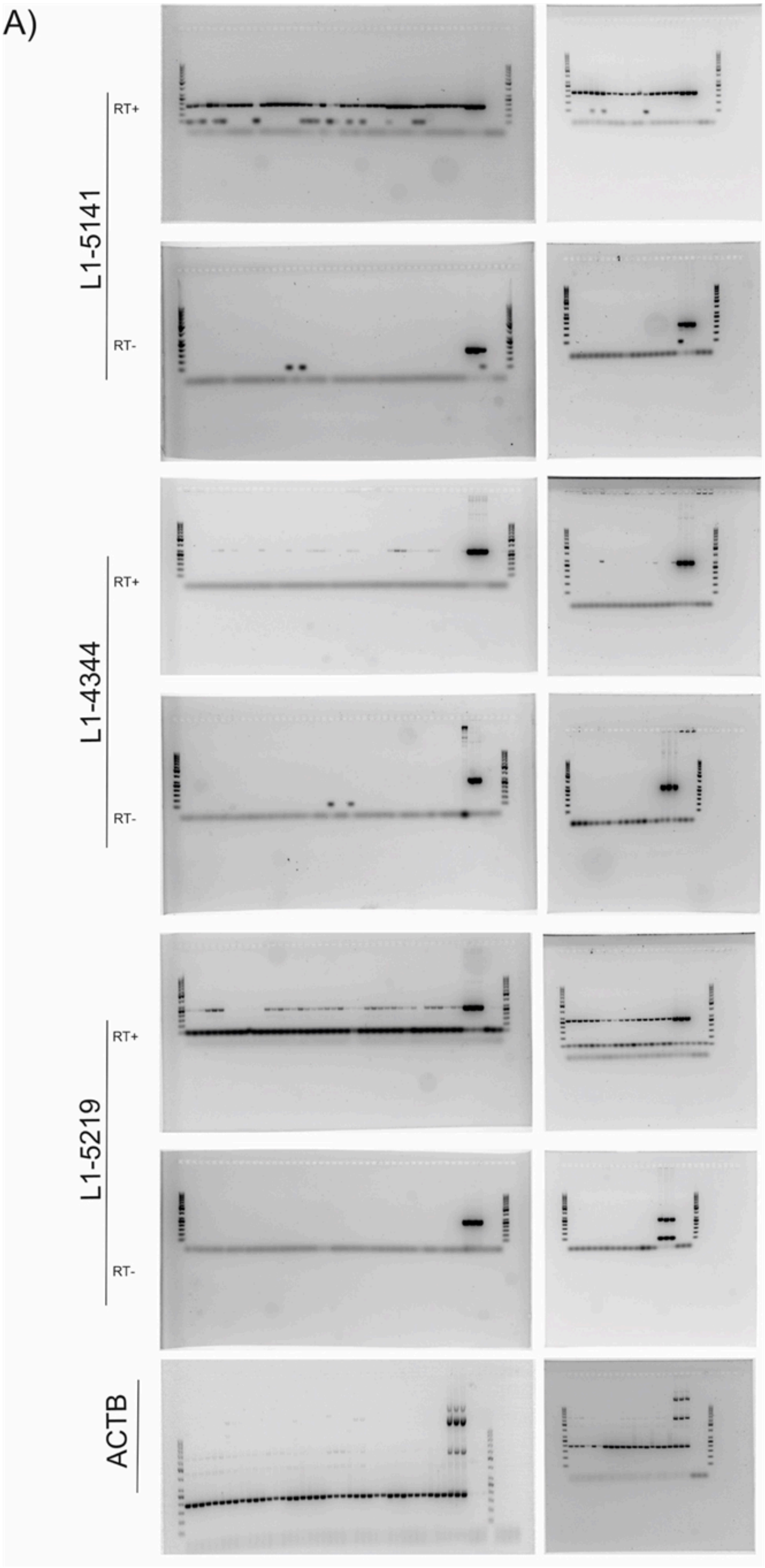
A)
L1-5141
RT+
RT-
L1-4344
RT+
RT-
L1-5219
RT+
RT-
ACTB

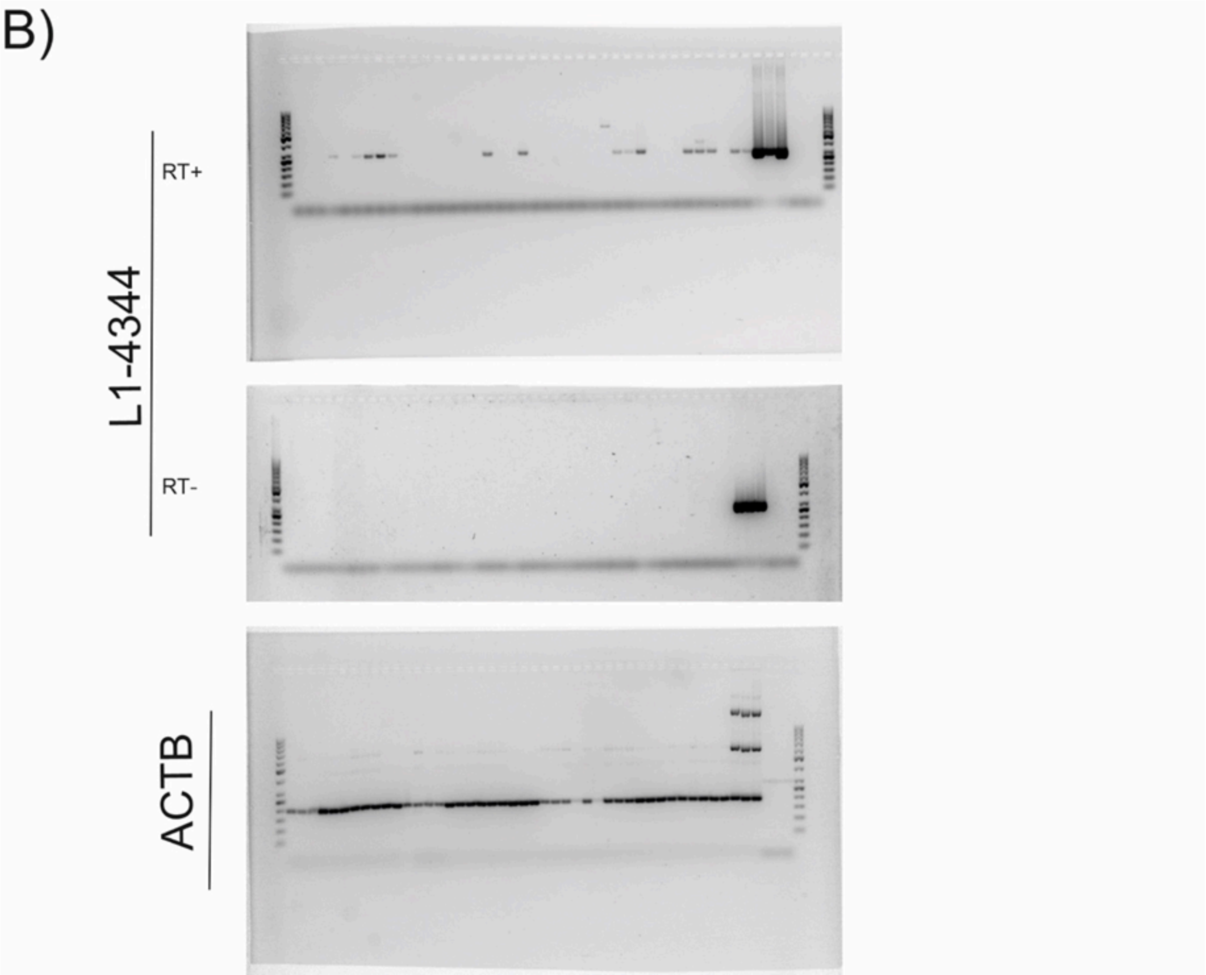

Supplementary Figure 4. *Uncropped images of agarose gels shown in A) Figure 3 and B) Supplementary Figure 5.*

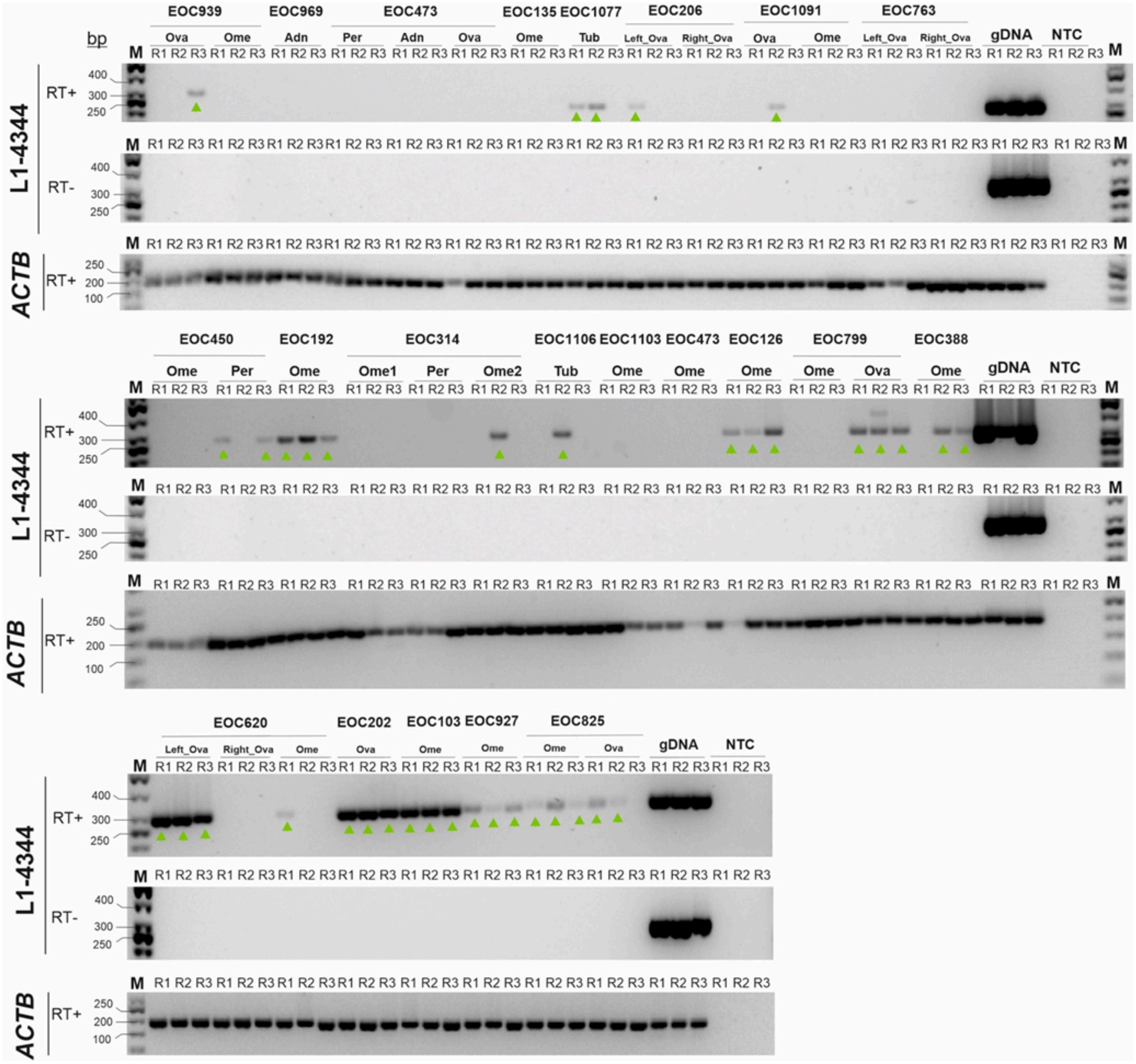

Supplementary Figure 5. *Locus-specific RT-PCR to detect expression of L1-4344 in an independent HGSOC patient cohort. Agarose gel image is shown with inverted colors (white-on-black) for ease of visualizing amplification products. RT-PCRs positive for amplification (L1-4344 RT-PCR product is sized 291 bp) are marked with green arrowheads. Genomic DNA was included as a control (lane labeled "gDNA"). RT-PCR with primers against ACTB transcript reports on the integrity of RNA samples. Abbreviations: R1,R2 & R3 = replicate 1, 2 & 3 respectively; p= primary (pre-chemotherapy); i= interval (post-chemotherapy); gDNA= genomic DNA; NTC= no template control; M= GeneRuler™ 50 bp DNA ladder, Thermo Scientific™); anatomical sites of tumor sample origin: Ova = ovary, Ome = omentum, Per = peritoneum, Adn = Adnexus, Tub = fallopian tube*

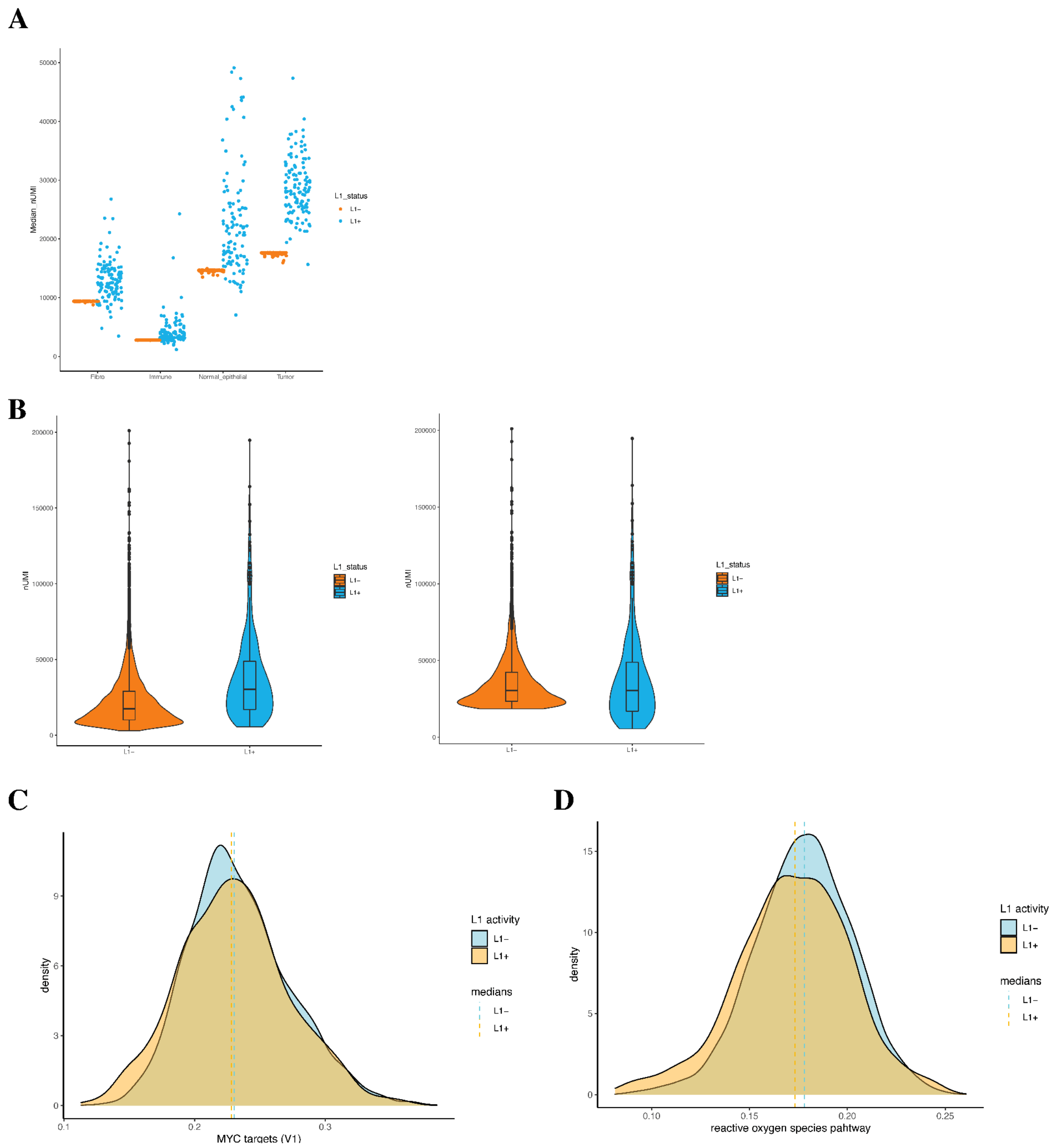

Supplementary Figure 6. UMI counts differ in L1+ and L1- cells. ***A.*** *Median UMI count in L1-positive and L1-negative cells (based on the validated L1 loci). One dot denotes a median UMI count in cells that do/do not express a particular L1.* ***B.*** *Cell size bias in L1-positivity. Number of UMIs in cancer cells that do (L1+) and do not (L1-) express the validated L1s, FL_L1-5219 and/or FL_L1-5141. C. Cell size bias in L1-positivity is reduced with the "median matching" procedure. Number of UMIs in subsetted cancer cells that do (L1+) and do not express (L1-) the validated L1s FL_L1-5219 and/or FL_L1-5141. Area Under the Curve (AUC) scores for "MYC targets"(**C**) and "Reactive oxygen species pathway" (**D**) hallmark gene sets between L1-positive and L1-negative cells. L1-positivity was assigned based on all the L1s appearing to be genuinely expressed in the scRNA-seq.*

Supplementary Table 1. *Percentiles of the total number of unique molecular identifiers (UMIs) per L1 locus, summed from all the samples of the dataset.*

0%	25%	50%	75%	95%	100%
1	1	4	12	95	7924

Supplementary Table 2. *Features of the top 5% most strongly expressed L1s of the scRNA-seq. Total UMI count = sum of UMIs from all the samples combined; Pearson's r/p.adj = Pearson's correlation and FDR adjusted p-value from the L1-host gene expression association analysis; Host expression enrichment in L1+ cells = In which cell types (if any) a host gene is upregulated in the resident L1-expressing cells compared to cells that do not express the resident L1; L1 expression high = Sample/cell types in which an L1 has a minimum of 3-fold enrichment in expression when compared to other sample/cell types; scRNA-seq N = Number of samples in which an L1 was active in scRNA-seq; Bulk RNA-seq N = Number of samples in which an L1 was active in bulk RNA-seq.*

L1 ID	location	Total UMI count	host gene(s)	Pearson's r/ p.adj (L1 vs. host)	Host expression enrichment In L1+ cells	L1 expression high	scRNA-seq N	Bulk RNA-seq N
Ewing2011-126	chr14:30679187-30683088	257	SCFD1 (antisense),	0.01/0.94	NA	NA	27	0
			UBE2CP1 (same-stranded)	NA/NA	NA			
Ewing2011-218	chr2:143250295-143254196	105	ARHGAP15 (same-stranded)	0.76/< 0.001	Yes (immune cells)	Yes (immune cells)	24	0
Ewing2011-230	chr2:190611556-190612556	158	NA	NA/NA	NA	NA	26	1
Ewing2011-369	chr6:13187873-13191774	130	PHACTR1 (same-stranded)	-0.14/0.29	Yes (tumor cells)	Yes (fibroblasts)	21	0
Ewing2011-58	chr10:120899751-120903652	253	WDR11 (same-stranded)	0.37/0.002	Yes (tumor cells, immune cells)	NA	25	2
FL-L1-0078	chr1:65449279-65457309	115	LEPR (antisense)	0.16/0.23	NA	Yes (tumor samples)	23	0
FL-L1-0132	chr1:85926070-85934100	144	COL24A1 (antisense)	-0.11/0.43	NA	Yes (tumor samples)	17	1
FL-L1-0193	chr1:113496220-113504257	281	MAGI3 (same-stranded)	0.65/< 0.001	Yes (tumor cells, immune cells)	NA	25	0
FL-L1-0233	chr1:149951052-149959158	899	OTUD7B (same-stranded)	0.29/0.02	Yes (tumor cells, fibroblasts, immune cells)	NA	27	0
FL-L1-0285	chr1:173724764-173732872	155	KLHL20 (same-stranded)	0.21/0.09	NA	NA	27	0

FL-L1-0289	chr1:174589352-174597377	229	RABGAP1L (antisense)	0.29/0.02	NA	Yes (normal samples)	23	0
FL-L1-0302	chr1:178836557-178844666	141	RALGPS2 (same-stranded)	0.63/< 0.001	NA	NA	24	1
FL-L1-0418	chr2:16592725-16600755	184	FAM49A (same-stranded)	0.65/< 0.001	NA	Yes (immune cells)	25	2
FL-L1-0457	chr2:40231600-40239627	201	SLC8A1 (same-stranded),	0.59/< 0.001	Yes (tumor cells, immune cells)	NA	26	0
			SLC8A1-AS1 (antisense)	NA/NA	NA			
FL-L1-0517	chr2:67188811-67196967	100	AC023115.2 (antisense),	0.17/0.21	NA	NA	22	0
			AC078941.1 (same-stranded)	0.50/< 0.001	NA			
FL-L1-0521	chr2:71410474-71418501	833	ZNF638 (antisense)	0.49/< 0.001	Yes (fibroblasts, immune cells)	NA	27	0
FL-L1-0585	chr2:110411395-110419526	153	AC108938.5 (same-stranded),	NA/NA	NA	Yes (normal samples, normal epithelial cells)	22	0
			RP13-1039J1.2 (same-stranded),	NA/NA	NA			
			RP13-1039J1.4 (same-stranded	NA/NA	NA			
FL-L1-0654	chr2:143005044-143013076	742	KYNU (same-stranded)	0.97/< 0.001	Yes (immune cells)	Yes (tumor samples, immune cells)	27	0
FL-L1-0690	chr2:157396599-157397599	150	NA	NA/NA	NA	Yes (immune cells)	23	0
FL-L1-0783	chr2:196904589-196912619	702	PGAP1 (antisense)	0.55/< 0.001	Yes (tumor cells, immune cells)	NA	27	0
FL-L1-0808	chr2:212053824-212061959	97	ERBB4 (antisense)	0.44/< 0.001	Yes (tumor cells)	NA	18	0

FL-L1-0815	chr2:216453724-216461842	113	SMARCAL1 (same-stranded)	0.05/0.78	NA	NA	24	0
FL-L1-0831	chr2:232148196-232156229	136	DIS3L2 (antisense)	0.25/0.05	Yes (immune cells)	NA	25	0
FL-L1-0950	chr3:51454920-51463072	544	DCAF1 (antisense)	0.22/0.08	NA	NA	27	0
FL-L1-0965	chr3:57475938-57484084	263	DNAH12 (antisense)	0.83/< 0.001	Yes (tumor cells, fibro cells)	Yes (normal samples, normal epithelial cells)	27	0
FL-L1-0976	chr3:60959425-60967566	129	FHIT (same-stranded)	0.45/< 0.001	NA	NA	21	0
FL-L1-1112	chr3:112730267-112731267	295	NA	NA/NA	NA	Yes (tumor samples, tumor cells)	18	0
FL-L1-1116	chr3:116202385-116210424	174	LSAMP (same-stranded)	0.48/< 0.001	Yes (fibroblasts)	Yes (tumor samples, fibroblasts)	21	0
FL-L1-1125	chr3:119562180-119563180	303	NA	NA/NA	NA	Yes (tumor samples, immune cells)	24	1
FL-L1-1139	chr3:125848810-125856962	470	ENPP7P4 (same-stranded),<br>RP11-379B18.5 (antisense)	NA/NA<br>NA/NA	NA<br>NA	NA	27	0
FL-L1-1148	chr3:132370232-132371232	130	NA	NA/NA	NA	NA	22	0
FL-L1-1166	chr3:136478078-136486101	217	STAG1 (same-stranded)	0.38/0.001	Yes (tumor cells, immune cells)	NA	27	1
FL-L1-1190	chr3:142474105-142482225	640	ATR (same-stranded)	0.72/< 0.001	NA	NA	27	0
FL-L1-1251	chr3:172658987-172667103	2176	NCEH1 (same-stranded)	0.79/ < 0.001	Yes (all cell types)	NA	27	0
FL-L1-1272	chr3:188371709-188379858	2004	LPP (antisense)	0.30/0.01	Yes (normal epithelial cells)	NA	27	0

FL-L1-1391	chr4:53327731-53335762	129	SCFD2 (antisense)	-0.06/0.67	NA	NA	23	0
FL-L1-1394	chr4:53465373-53473500	189	LNX1 (antisense),	-0.002/0.98	Yes (tumor cells)	NA	25	1
			RP11-231C18.3 (same-stranded)	NA/NA	NA			
FL-L1-1468	chr4:72513834-72521993	111	ADAMTS3 (same-stranded)	0.15/0.27	Yes (fibroblasts)	Yes (fibroblasts)	17	0
FL-L1-1501	chr4:80668983-80677169	301	C4orf22 (antisense)	0.79/< 0.001	Yes (normal epithelial cells, fibroblasts)	Yes (normal samples, normal epithelial cells)	24	0
FL-L1-1532	chr4:90478376-90486523	95	CCSER1 (antisense)	0.72/< 0.001	Yes (tumor cells)	Yes (normal epithelial cells)	20	0
FL-L1-1568	chr4:98591435-98599461	97	TSPAN5 (same-stranded)	0.03/0.86	Yes (immune cells)	NA	24	0
FL-L1-1594	chr4:105879181-105887196	198	INTS12 (antisense)	-0.08/0.57	NA	Yes (normal epithelial cells)	25	1
FL-L1-1644	chr4:122938633-122946796	257	SPATA5 (antisense)	0.09/0.54	Yes (immune cells)	NA	27	0
FL-L1-1718	chr4:151510300-151518459	359	FAM160A1 (same-stranded)	0.39/< 0.001	NA	NA	25	0
FL-L1-1867	chr5:42937866-42938866	254	NA	NA/NA	NA	Yes (normal samples)	21	0
FL-L1-1872	chr5:43666275-43674422	464	NNT (antisense),	0.11/0.44	NA	NA	27	0
			RPL29P12 (same-stranded)	0.94/< 0.001	Yes (fibroblasts)			
FL-L1-1998	chr5:78434233-78442331	155	SCAMP1 (antisense)	0.03/0.86	NA	NA	21	0
FL-L1-2050	chr5:93760070-93768199	820	FAM172A (antisense)	0.23/0.06	Yes (fibroblasts, immune cells)	NA	27	0
FL-L1-2121	chr5:119438179-119446196	137	RNA5SP190 (same-stranded)	0.99/< 0.001	NA	Yes (tumor samples, fibroblasts)	21	0

FL-L1-2166	chr5:132519996-132520996	140	NA	NA/NA	NA	NA	24	4
FL-L1-2194	chr5:150741837-150749950	190	DCTN4 (antisense)	-0.04/0.79	NA	NA	25	0
FL-L1-2276	chr6:12138368-12146531	169	HIVEP1 (same-stranded)	0.27/0.03	Yes (tumor cells)	NA	27	6
FL-L1-2293	chr6:24810678-24818704	514	FAM65B (same-stranded)	0.89/< 0.001	Yes (immune cells)	Yes (normal samples)	26	1
FL-L1-2310	chr6:44869634-44877665	180	AL161905.1 (antisense),<br>SUPT3H (antisense)	NA/NA<br>0.29/0.02	NA<br>NA	NA	24	0
FL-L1-2564	chr6:116637699-116645834	111	ZUFSP (antisense)	0.16/0.21	NA	NA	25	0
FL-L1-2677	chr7:22923196-22931356	296	FAM126A (antisense)	0.57/< 0.001	Yes (fibroblasts, immune cells)	NA	27	0
FL-L1-2690	chr7:30438244-30446274	878	NOD1 (antisense)	0.23/0.07	Yes (immune cells)	NA	27	0
FL-L1-2712	chr7:36551968-36560126	107	AOAH (same-stranded)	0.76/< 0.001	Yes (immune cells)	Yes (normal samples, immune cells)	17	2
FL-L1-2763	chr7:70196331-70204357	139	AUTS2 (same-stranded)	0.04/0.78	NA	NA	23	0
FL-L1-2776	chr7:83988325-83996342	552	SEMA3A (same-stranded)	0.85/< 0.001	Yes (tumor cells, fibroblasts)	NA	24	0
FL-L1-2842	chr7:108386569-108394721	118	NRCAM (same-stranded)	0.56/< 0.001	NA	NA	18	0
FL-L1-2890	chr7:130576798-130584457	96	COPG2 (antisense)	0.15/0.25	NA	Yes (tumor samples)	23	0
FL-L1-2903	chr7:140102318-140110394	1049	KDM7A (same-stranded)	0.40/< 0.001	Yes (fibroblasts, immune cells)	NA	27	0
FL-L1-2919	chr7:147841591-147849620	159	CNTNAP2 (antisense)	-0.008/0.95	NA	NA	8	3

FL-L1-2956	chr8:15555008-15563026	222	TUSC3 (antisense)	0.48/< 0.001	NA	NA	26	0
FL-L1-2974	chr8:26394612-26402579	542	BNIP3L (same-stranded)	0.09/0.55	Yes (immune)	NA	27	0
FL-L1-3027	chr8:47483871-47492006	429	SPIDR (same-stranded)	0.56/ < 0.001	Yes (immune cells)	NA	27	0
FL-L1-3030	chr8:48333021-48334021	294	NA	NA/NA	NA	Yes (tumor samples)	12	0
FL-L1-3047	chr8:53779543-53787666	802	ATP6V1H (same-stranded)	0.55/< 0.001	Yes (tumor cells, immune cells)	NA	27	0
FL-L1-3048	chr8:54579988-54588142	207	RP1 (antisense)	0.84/< 0.001	Yes (tumor cells)	Yes (normal samples, normal epithelial cells)	26	0
FL-L1-3194	chr8:98693365-98701518	1114	STK3 (same-stranded)	0.71/< 0.001	Yes (fibroblasts)	NA	27	0
FL-L1-3215	chr8:105788089-105796234	370	ZFPM2 (antisense),	-0.01/0.94	NA	NA	21	0
			ZFPM2-AS1 (same-stranded)	NA/NA	NA			
FL-L1-3238	chr8:118530003-118537089	128	SAMD12 (same-stranded)	0.60/< 0.001	Yes (tumor cells)	Yes (tumor samples, tumor cells)	24	3
FL-L1-3248	chr8:120643161-120651187	100	SNTB1 (same-stranded)	0.28/0.02	NA	NA	24	0
FL-L1-3307	chr9:5030773-5038865	334	JAK2 (same-stranded)	-0.03/0.86	NA	NA	26	1
FL-L1-3310	chr9:5721562-5729670	708	RIC1 (antisense)	-0.04/0.80	Yes (immune cells)	NA	27	0
FL-L1-3345	chr9:21818140-21826239	1295	MTAP (antisense),	0.21/0.10	Yes (immune cells)	NA	27	0
			RP11-145E5.5 (antisense)	NA/NA	NA			
FL-L1-3437	chr9:83048539-83056569	235	RASEF (same-stranded)	0.64/< 0.001	NA	NA	21	1
FL-L1-3566	chr10:35472782-35480771	325	CCNY (same-stranded)	0.14/0.30	NA	NA	27	0

FL-L1-3636	chr10:74215882-74224021	384	ADK (same-stranded)	-0.08/0.56	Yes (fibroblasts)	NA	27	4
FL-L1-3645	chr10:78094479-78095479	136	NA	NA/NA	NA	NA	21	1
FL-L1-3670	chr10:87896804-87904979	1292	PTEN (antisense)	0.25/0.04	Yes (immune cells)	NA	27	0
FL-L1-3721	chr10:111049281-111050281	246	NA	NA/NA	NA	Yes (tumor samples)	25	0
FL-L1-3741	chr10:129731560-129739580	107	MGMT (antisense)	0.24/0.05	NA	Yes (normal samples)	20	0
FL-L1-3767	chr11:9539333-9540333	127	NA	NA/NA	NA	NA	21	0
FL-L1-3778	chr11:14660329-14668447	178	PDE3B (same-stranded)	0.83/< 0.001	Yes (immune cells)	Yes (immune cells)	24	3
FL-L1-3779	chr11:14714908-14722938	181	PDE3B (same-stranded)	0.78/< 0.001	Yes (immune cells)	Yes (immune cells)	23	6
FL-L1-3780	chr11:14776675-14784773	521	PDE3B (same-stranded)	0.93/< 0.001	Yes (immune cells)	Yes (immune cells)	26	8
FL-L1-3945	chr11:71510047-71518198	96	NADSYN1 (same-stranded)	0.31/0.01	NA	NA	22	0
FL-L1-3949	chr11:74886437-74894565	153	RP11-147I3.1 (same-stranded),<br>XRRA1 (antisense)	NA/NA<br>0.02/0.88	NA<br>NA	NA	25	2
FL-L1-3997	chr11:93171447-93179521	178	RP11-573M3.2 (antisense),<br>SLC36A4 (same-stranded)	NA/NA<br>0.27/0.02	NA<br>NA	NA	23	0
FL-L1-4034	chr11:107371762-107379780	213	CWF19L2 (antisense)	0.38/0.001	NA	Yes (normal samples)	26	0
FL-L1-4122	chr12:26668557-26676709	179	ITPR2 (antisense)	0.18/0.15	NA	NA	27	0
FL-L1-4311	chr12:96314944-96322971	451	CDK17 (antisense)	0.42/< 0.001	Yes (immune cells)	NA	27	0
FL-L1-4344	chr12:126298010-126306038	103	RP4-809F18.1 (antisense)	NA/NA	NA	Yes (tumor samples, fibroblasts)	10	0

FL-L1-4371	chr13:29822715-29830837	337	UBL3 (same-stranded)	0.39/< 0.001	Yes (tumor cells)	NA	27	0
FL-L1-4405	chr13:50179295-50187327	174	DLEU1 (antisense)	0.35/0.003	Yes (immune cells)	Yes (tumor samples)	24	3
FL-L1-4598	chr14:56227636-56235747	198	PELI2 (same-stranded)	0.33/0.006	Yes (immune cells)	NA	27	0
FL-L1-4634	chr14:70546296-70554322	176	RP11-486O13.4 (antisense)	NA/NA	NA	NA	21	0
FL-L1-4719	chr15:52471398-52479553	496	MYO5A (same-stranded)	0.62/< 0.001	Yes (immune cells)	Yes (immune cells)	27	1
FL-L1-4731	chr15:56133618-56141628	142	RFX7 (same-stranded)	0.17/0.18	NA	NA	25	2
FL-L1-4738	chr15:64325660-64333700	7924	CSNK1G1 (antisense),	0.32/0.009	Yes (normal epithelial cells, fibroblasts)	NA	27	0
			CTD-2116N17.1 (antisense)	NA/NA	NA			
FL-L1-4766	chr15:85432963-85440965	156	AKAP13 (same-stranded)	-0.05/0.78	Yes (tumor cells)	NA	27	1
FL-L1-4792	chr15:100082863-100090998	223	ADAMTS17 (antisense)	0.25/0.04	Yes (immune cells)	NA	26	0
FL-L1-4811	chr16:31344038-31345038	95	NA	NA/NA	NA	Yes (tumor samples)	19	0
FL-L1-4815	chr16:31943726-31944726	113	NA	NA/NA	NA	NA	19	0
FL-L1-4886	chr16:72579798-72587932	212	LINC01572 (antisense)	0.21/0.10	NA	NA	27	0
FL-L1-4939	chr17:66595579-66603592	802	PRKCA (antisense)	0.10/0.47	Yes (immune cells)	Yes (tumor samples)	24	0
FL-L1-4968	chr18:13342089-13350216	146	LDLRAD4 (antisense)	0.04/0.79	NA	NA	26	0
FL-L1-5023	chr18:49101418-49109547	293	DYM (same-stranded)	0.11/0.43	NA	NA	26	10
FL-L1-5063	chr19:12003811-12011812	104	CTD-2006C1.2 (antisense)	NA/NA	NA	NA	26	3
FL-L1-5102	chr19:40036915-40045054	566	ZNF780B (same-stranded)	0.04/0.82	Yes (immune cells)	NA	27	1

FL-L1-5141	chr20:23432140-23433140	146	NA	NA/NA	NA	Yes (tumor samples, tumor cells)	21	16
FL-L1-5154	chr20:35070283-35078389	501	TRPC4AP (same-stranded)	0.35/0.003	Yes (immune cells)	NA	27	0
FL-L1-5210	chr22:23661983-23670138	2336	AP000347.2 (antisense),	0.65/< 0.001	Yes (immune cells)	NA	27	0
			GUSBP11 (antisense),	NA/NA	NA			
			KB-1572G7.2 (antisense)	NA/NA	NA			
FL-L1-5219	chr22:28662285-28670315	160	TTC28 (antisense)	-0.05/0.78	NA	Yes (tumor samples, tumor cells)	18	18
Helman2014-185	chr18:72844266-72848167	642	NETO1 (same-stranded)	0.94/< 0.001	Yes (fibroblasts)	Yes (tumor samples, fibroblasts)	17	3
Lee2012-414	chr7:77091735-77095636	323	FAM185BP (antisense),	0.27/0.03	NA	NA	27	0
			RP11-467H10.2 (same-stranded)	NA/NA	NA			
Schwahn1998-464	chrX:46834934-46838835	211	RP2 (same-stranded)	0.11/0.43	Yes (immune cells)	NA	26	24
Shukla2013-141	chr14:94173729-94177630	171	PPP4R4 (same-stranded)	0.65/< 0.001	Yes (fibroblasts)	NA	25	4
Shukla2013-194	chr2:36389671-36393572	1037	CRIM1 (same-stranded)	0.50/< 0.001	Yes (tumor cells, immune cells)	NA	27	1
Shukla2013-41	chr10:7980124-7984025	145	TAF3 (same-stranded)	0.27/0.03	NA	NA	25	0
Shukla2013-425	chr7:135157267-135161168	814	C7orf49 (antisense),	-0.11/0.43	NA	Yes (immune cells)	27	23
			TMEM140 (same-stranded)	0.24/0.06	Yes (tumor cells, immune cells)			
Shukla2013-85	chr11:117322084-117325985	173	AP000892.4 (same-stranded),	NA/NA	NA	NA	26	0
			CEP164 (antisense)	0.14/0.30	NA			

Supplementary Table 3. *Primers used for RT-PCR.*

**Target locus**	**Primer name**	**Sequence (5'-3')**
L1-5141	L1_008 (forward)	AGATATACCTAATGCTAGATGACACA
L1-5141	L1_54A (reverse)	GCATCTTATCGACTGGTGCATG
L1-5219	L1_008 (forward)	AGATATACCTAATGCTAGATGACACA
L1-5219	L1_003 (reverse)	ATTTTGGCCCTAACTGGTCACA
L1-4344	L1_008 (forward)	AGATATACCTAATGCTAGATGACACA
L1-4344	L1_4344_dR2 (reverse)	AACATGTCAACCTGCAGAACC
ACTB	MT_126 (forward)	AGAAAATCTGGCACCACACC
ACTB	MT_127 (reverse)	AGAGGCGTACAGGGATAGCA
